# Aniline dimers serving as stable and efficient transfer units for intermolecular charge-carrier transmission

**DOI:** 10.1016/j.isci.2022.105762

**Published:** 2022-12-08

**Authors:** Juexin Huang, Chuanliang Feng

**Affiliations:** 1 State Key Lab of Metal Matrix Composites, School of Materials Science and Engineering, Shanghai Jiao Tong University, 800 Dongchuan Road, Shanghai 200240, P. R. China

**Keywords:** Organic chemistry, applied sciences

## Abstract

Because any perturbation in the number of oxidation sites associated with the polymeric backbone can cause changes in the electrical properties, the stability of electrical properties has strongly prevented the wide adoption of most conducting polymers for commercialization, e.g., polyanilines (PANI). Herein, we showed that aniline dimers (AD) had more stable conductivity during redox due to their determinately separate oxidization or reduction units. Instead of intramolecular charge transfer as PANI, AD could serve as effective transfer units to facilitate intermolecular charge-carrier transmission due to low band-gap formation induced by the J-aggregation of AD, ensuring efficient conductivity. Typically, the electrical properties of AD-derived materials will still be stable after 10,000 redox cycles under a high operating voltage, far surpassing PANI under equivalent conditions. Meanwhile, the AD-derived materials could act as effective conducting and sensing layers with good stability. This approach opened an avenue for improving the stability of conductive polymers.

## Highlights

• J-aggregated aniline dimers (AD) units ensure stable and efficient intermolecular charge-carrier transmission
• AD-derived materials will still be stable after 10,000 redox cycles, far surpassing polyanilines
• AD-derived materials could act as effective conducting and sensing layers

## Graphical abstract

**Figure:**
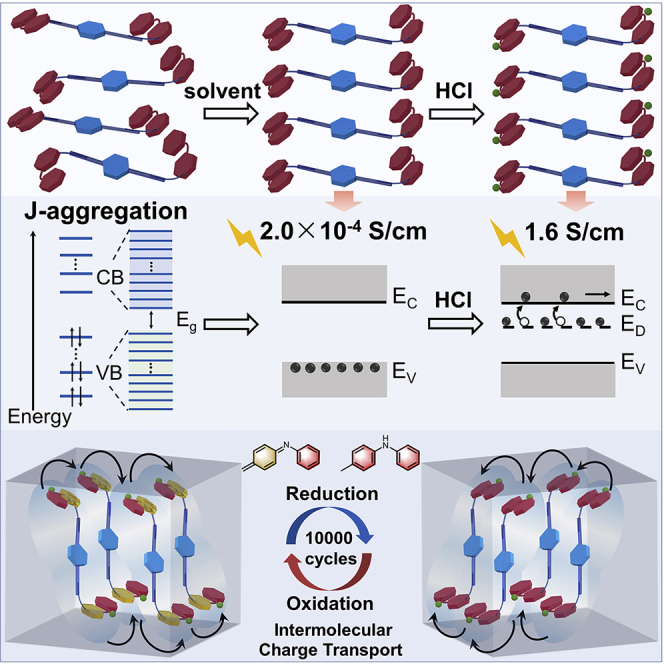

## Introduction

Inherently conducting polymers (ICPs) have reasonable potential to replace various inorganic materials such as semiconductors and metal materials due to lower manufacturing costs, lower density, better processability, higher mechanical flexibility, and broader chemical functionalization capabilities.^1^ Among all ICPs, polyanilines (PANI) are considered the least expensive and the most thermostable. They also have tunable conductivity^2^ and have been widely used in sensors,^3^^,^^4^^,^^5^^,^^6^ secondary batteries,^7^ catalysts,^8^ and other areas,^9^^,^^10^^,^^11^^,^^12^ leading to the supposition that they may be the best ICP.^13^ However, limitations in the stability of electrical properties have prevented the wide commercialization use of PANI, as their electrical properties through intramolecular charge transfer are usually unstable due to abundantly localized states and traps induced by the indeterminacy of oxidation sites and inhomogeneous doping,^14^^,^^15^^,^^16^ These are considered inherent properties of PANI, and the electrical properties can be greatly varied with further oxidization or reduction. To improve stability, the majority of preliminary reports have focused on enhancing doping stability,^17^^,^^18^^,^^19^^,^^20^ which has successfully expanded the application range of PANI. However, employing doping to maintain PANI in a conductive state (emeraldine salt) requires the stable backbone structures of PANI, and any perturbation in the number of oxidation sites associated with the polymeric backbone will cause changes in electrical properties. Therefore, it is a great challenge to reduce the effects of the intrinsic redox states in the chains on the electrical properties.

The conductive mechanism of PANI should obey “polaron lattice model”^21^ and “four ring BQ derivatives.”^22^ In the mechanism, the PANI chains contained both reduction and oxidization repeat units, and if these two parts were equal in ratio, the highest conductivity was expected after doping. Nevertheless, the ratio was quite fragile for the redox process (Figure S1), as PANI generally suffers from long-term storage,^23^ in-service environments with voltage changes^24^^,^^25^^,^^26^ or radiation.^27^ In this case, the efficiency of intramolecular charge transfer would directly influence and further lead to unstable conductivity, resulting in obvious weakness for PANI or oligoanilines when acting as conductive materials with high-performance. Therefore, breaking the dependence of electrical PANI properties on intramolecular charge transport was shown to be the key to solve the problem, and a change in the concept toward the conduction mechanism was required to improve the stability of PANI.

**Figure 1:**
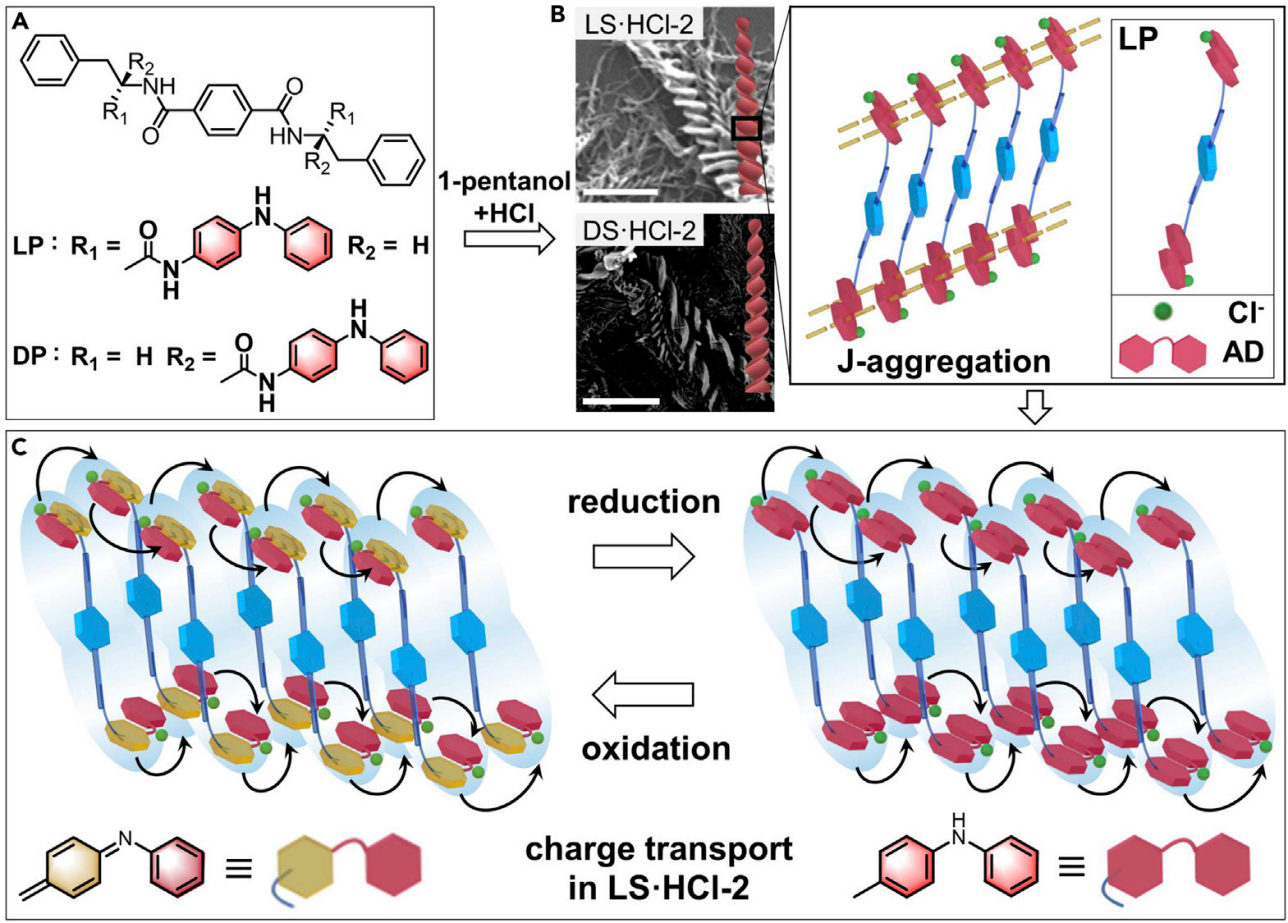
Morphologies and intermolecular charge-carrier transmission of LP and DP assemblies (A) Chemical structures of the AD units symmetrically coupled on the *C*_2_-symmetric structure with L- or D-phenylalanine arms (LP or DP). (B) SEM images (scale bars: 2 μm) and J-aggregation of the LP and DP assemblies in the 1-pentanol+HCl mixture solution. The top SEM image shows the LP assemblies (denoted as LS·HCl-2); while the bottom SEM image shows the DP assemblies (denoted as DS·HCl-2), with AD indicated by the red blocks, and Cl^−^ indicated by the green spheres. (C) Schematic illustration of the charge transport in LS·HCl-2 during redox, with AD in the reduction state indicated by the red blocks, AD in the oxidation state indicated by the khaki and red blocks; Cl^−^ indicated by the green spheres; and the shaded blue regions indicating the spatial extent of the carrier wavefunction.

To minimize the effect of intrinsic redox states in the chains on the electrical properties, herein, AD units were symmetrically coupled on a well-developed *C*_2_-symmetric structure^28^ with L- or D-phenylalanine arms (denoted as LP or DP) (Figure 1A). During redox, LP or DP exhibited determinately separate oxidization or reduction AD units, which avoided localized states and traps, caused by disordered molecular structures.^14^^,^^15^^,^^16^ Meanwhile, their efficient self-assembly ability led to the J-aggregation formation of the AD units in the 1-pentanol-HCl solvents, resulting in low band-gap formation in the nanostructures. Moreover, HCl doping could further extend the electron delocalization of the assemblies, providing free electrons for the conduction band and ensuring the macroscopic conduction of the nanostructures. This allowed the AD segments to act as stable and efficient transfer units to fulfill intermolecular charge-carrier transmission in the materials Figure 1C), which was a different charge transfer mechanism compared to PANI and other oligoanilines. The conductivity of LP reached ∼0.127 S/cm in the bulk state and ∼1.603 S/cm in the single nanofiber, which was ∼4.4 times higher than that of the single PANI nanosheet (0.362 S/cm). During redox under a high constant potential in neutral aqueous solutions, the variation in charge transfer resistance (R_CT_) for LP was less than 36.4% and more than 240% for PANI, and excellent cyclic stability during 10,000 cycles of LP was observed, far surpassing PANI under equivalent conditions.

## Results and discussion

### Intermolecular arrangements and narrow band-gap of L-phenylalanine assemblies

LP and DP were synthesized by a conventional liquid-phase reaction in high yields (Figures S2–S7). They can self-assemble into sea-urchin- such as structures in hexafluoroisopropanol (HFIP) (Figures S14A and S14B) and nanofibers in HFIP-HCl (Figures S14C and S14D). In 1-pentanol-HCl, left-handed (M-type) or right-handed (P-type) helicity with a diameter of hundreds of nanometers are observed for LP or DP, respectively (Figures 1B, S14E, and S14F). Both LP and DP show light purple color in HFIP (denoted as LS-1, DS-1), grayish green in HFIP-HCl (denoted as LS·HCl-1, DS·HCl-1), and grayish black in 1-pentanol-HCl (denoted as LS·HCl-2 and DS·HCl-2) (Figures S14A, S14C, S14E and S14F, inset), respectively.

The intermolecular arrangements of LS-1, LS·HCl-1, and LS·HCl-2 were studied by UV-Vis spectra. The absorption peak of LS·HCl-2 shows a blue shift from 293 to 270 nm with decreasing concentration of LP (Figure S15A), which is related to the blue shift of the absorption peak of AD units. Originally, the absorption peak of AD molecules should locate at ∼298 nm in 1-pentanol-HCl and no peak shift appears with varying concentrations (Figure S15B). Firstly, AD units are linked to the electron-withdrawing group C=O in LP, thus, the decreased electron density in AD is expected and the blue shift of absorption peak is observed. Secondly, blue shift from 293 to 270 nm suggests that π-π interaction between AD units is decreased, corresponding to the disassociation of J-aggregation^29^^,^^30^ of AD units. LP in 1-pentanol (denoted as LS-2) has similar phenomena (Figure S15D). Here, no peak shift at ∼ 298 nm for LS-1 and LS·HCl-1 (Figures S15E and S15F) implies that 1-pentanol plays a key role in J-aggregation formation of AD units. Meanwhile, the extent of LS·HCl-2 self-assembly can be monitored by absorbance, as J-aggregation formation results in the spectral centroid continue to shift toward lower energies in J-aggregates that favors excitation to the lowest vibrational level (0-0) of the excited state instead of the second vibrational level (0-1). This results in a diagnostic change in the Franck-Condon factors,^31^^,^^32^ which is manifested by a dramatic alteration in the ratio of the absorbance vibrational peaks R_abs_ = A^0–0^/A^0-1^. A Gaussian deconvolution of the UV-vis absorbance reveals an increase of R_abs_. (Figures S15G–S15K), claiming J-aggregation formation of AD units.

**Figure 2:**
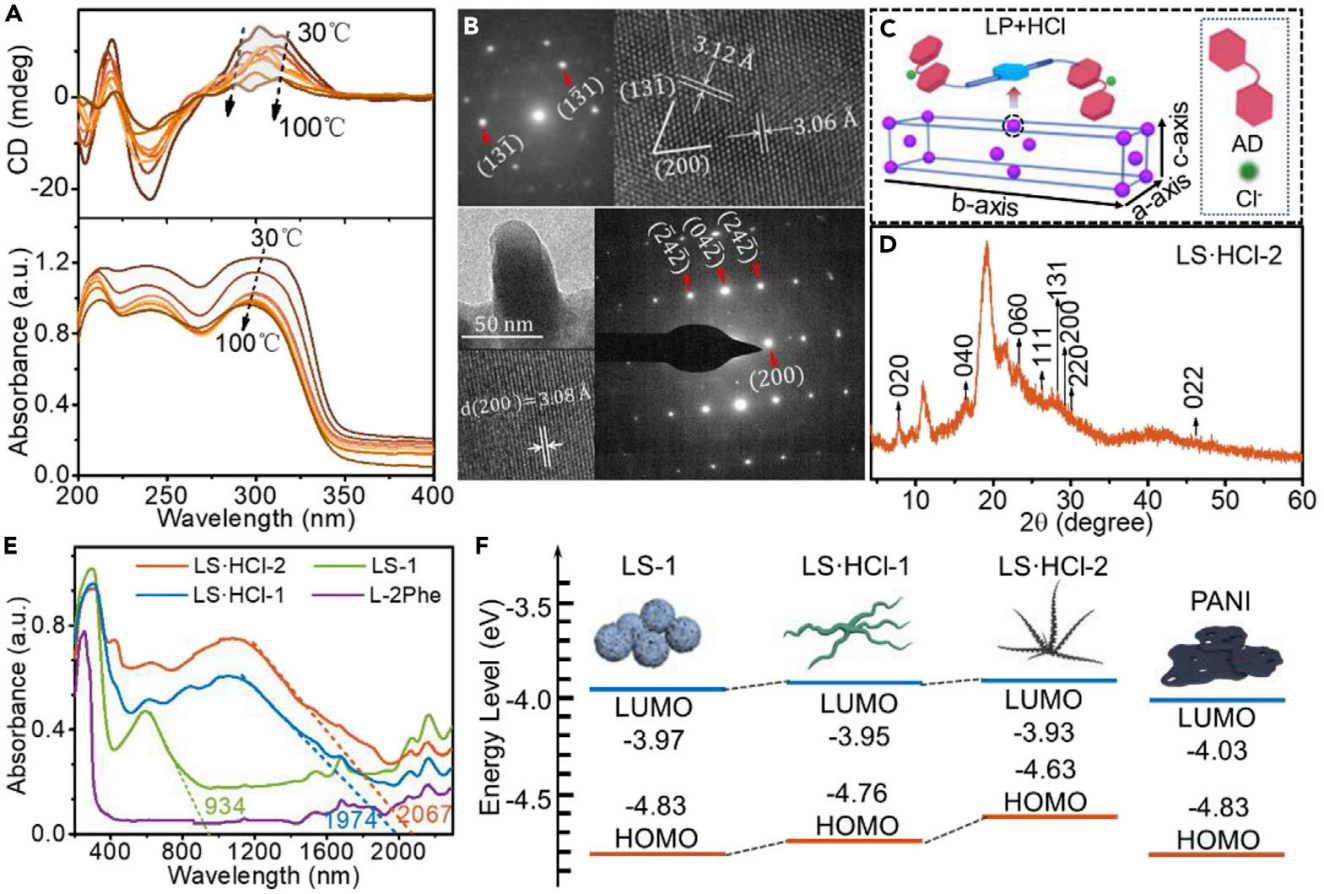
Structures and physical properties of the LP assemblies (A) Dynamic CD spectra and variable temperature UV spectra of LS·HCl-2. (B) SAED patterns for LS·HCl-2. The insets show the corresponding bright-field TEM images. (C) The unit cell representation of LS·HCl-2 from SAED. The purple ball indicated an LP molecule; AD is indicated by a red block; Cl^−^ is indicated by a green sphere. (D) Powder X-ray diffraction (XRD) patterns of LS·HCl-2. (E) Diffuse reflectance UV-Vis-NIR spectroscopy of LS-1, LS·HCl-1 LS·HCl-2, and L-2Phe. (F) Highest unoccupied molecular orbital (HOMO) and lowest unoccupied molecular orbital (LUMO) energy levels of LS-1, LS·HCl-1, LS·HCl-2, and PANI.

By circular dichroism (CD), a red-shift of maximum CD signals from 271 to 275 nm is observed with increasing solution concentration of LS·HCl-2 (Figure S16A), further claiming J-aggregation formation of AD units. The peak value of anisotropy factor (g-factor) is ∼22 times and 2 times higher than that of LS-1 and LS·HCl-1 in the region of 260-330 nm (Figure S16B), respectively, ascribing to the enhanced anisotropy and in turn regularity of LS·HCl-2. DS·HCl-2 also shows a stronger chiral signal than those of DS-1 and DS·HCl-1 (Figure S16C). By temperature-dependent CD spectra from 30 °C to 100 °C, the positive CD peak at 302 nm gradually decreases and blue shifts to 296 nm until complete disappearance due to the disassociation of J-aggregation (Figure 2A).

The packing structures of LS-1, LS·HCl-1, and LS·HCl-2 were probed by transmission electron microscopy (TEM) (Figures 2B and S17A–S17H). The sharp Bragg spots indicate a good crystallinity of LS·HCl-2 nanofibers. The selected area electron diffraction (SAED) pattern and high-resolution TEM image suggest a (200) d-spacing of ∼3.06 Å for LS·HCl-2 (Figure 2B). A packing model having a set of (100), (010), and (001) d-spacing of 6.14, 22.32, and 4.07 Å was proposed (Figure 2C). By powder X-ray diffraction (XRD), the crystal structure of LS·HCl-2 has the d-spacing of 11.16, 5.46, and 3.76 Å, in agreement with (020), (040), (060) spacing, respectively (Figure 2D). Here, the strong peak intensity of XRD at ∼20° is indicative of the π-stacked aromatics.^33^ The diffraction of LS·HCl-2 appearing at higher degrees (2θ = 19.33°; d = ∼4.58 Å) than that of LS-1 or LS·HCl-1 suggests a narrower distance between aromatic planes (Figure S19A). While, wider diffraction peaks for non-AD units coupled *C*_2_-symmetric molecules (L-2Phe) (Chemical structures in Figure S16E) and PANI, implying a lower crystallinity index for them (Figure S19A).

The driving force for the regular structure formation of LS·HCl-2 was detected by Fourier transform infrared spectroscopy (FT-IR) and Fluorescence spectrum. By FT-IR, two IR bands at 3447-3373 cm^−1^ and 3340-3220 cm^−1^ appear for LS-1, LS·HCl-1, and LS·HCl-2, owing to the stretching vibrations of N-H bonds (νNH). The νNH of LS·HCl-2 (∼3385 and ∼3269 cm^−1^) shows a red-shift compared with those of LS-1 and LS·HCl-1 (Figure S19B) because of the stronger intermolecular interactions.^34^ The fluorescence intensity of LS·HCl-2 in the solid state shows a considerable decrease than that in solution (Figure S20A), further claiming the stronger intermolecular π-π stacking interactions.^35^ While the largest Stokes shift for LS·HCl-2 among all LP assemblies (Figure S20C) indicates a more profound structural and energetic relaxation process in an excited state.^36^^,^^37^

The well-defined nanostructures can induce intermolecular hybridization and facilitate the formation of low band-gap. By diffuse reflectance ultraviolet-visible-near-infrared (UV-Vis-NIR) spectra (Figure 2E), the absorption onset is at ∼934 nm for LS-1, whereas red-shifts to ∼1974 and ∼2067 nm for LS·HCl-1 and LS·HCl-2, respectively, suggesting lower band-gap for LS·HCl-1 and LS·HCl-2 (Figure 2E). The optical energy gap calculated by Kubelka-Munk function decreased from 1.48, 0.71 to 0.66 for LS-1, LS·HCl-1, LS·HCl-2 (Figure S21A). The electrochemical gaps by cyclic voltammetry (CV) also decrease from 0.86, 0.81, to 0.7 eV for them (Figures 2F and S22A–S22D). The narrowing band-gap should ascribe to the upward shift of highest occupied molecular orbital (HOMO) (Figure 2F) from LS-1, LS·HCl-1 to LS·HCl-2. By density functional theory (DFT), HOMO should localize on AD units and phenylalanine motifs have a little contribution to HOMO (Figures S23A–S23C).

### Effect of HCl doping on the energy band structure of L-phenylalanine assemblies

**Figure 3:**
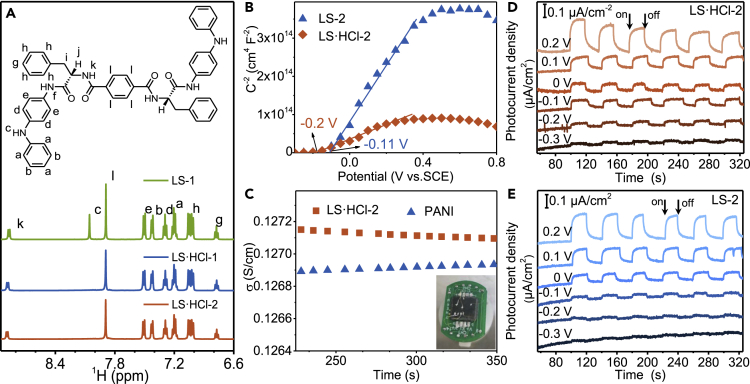
The electrical property and flat band potential of LS·HCl-2 (A) ^1^H NMR spectrum of LP assemblies in DMSO-dDMSO-*d*_*6*_ solution. A sharp singlet at 8.06 ppm (2H_z,__c_) for LS-2 ascribes to the secondary amine (N-H) protons of AD. The peak disappears for LS·HCl-2 and LS·HCl-1 due to the formation of active hydrogen with HCl. (B) Mott-Schottky plot of LS·HCl-2 and LS-2 in 0.2 M KCl solution at a frequency of 5 kHz. (C–E) Electrical conductivity of pressed-pellet PANI and LS·HCl-2. Inset shows the photograph of the typical LS·HCl-2 sample. (D and E) photoresponsive current of (D) LS·HCl-2 and (E) LS-2 (obtained from1-pentanol) at different voltage bias under the illumination of 365 nm.

After HCl doping, -NHPh group in AD can be acidified as detected by ^1^H NMR and high-resolution X-ray photoelectron spectroscopy (Figures 3A and S27A). By Mott-Schottky (M−S) analysis, linear relationships of 1/C^2^ vs V in the range of −0.6-0.8 V with positive slopes suggests n-type semiconductor^38^^,^^39^ for LS·HCl-2 and LS-2 (Figure 3B). Much small slope for LS·HCl-2 ascribes to the high charge carrier density^40^ after HCl doping. According to flat band potential (V_fb_) computed by X axis intercept of 1/C^2^ vs V, V_fb_ of LS·HCl-2 is −0.2 V and −0.11 V for LS-2, suggesting Fermi level shifts upward^41^ after AD protonation for LS·HCl-2 (Figure 3B). The photoresponsibility of LS·HCl-2 remains significant even though the bias voltage decreases to −0.4 V, while it almost drops to zero for LS-2 (Figures 3D, 3E, and S28A), agreeing well with upward shift of the Fermi level. From DFT calculation, Cl^−^ can act as an electron donor to transfer their charge to AD units in LS·HCl-2 (Figures S23B and S23C) as reported.^42^ Together with AD J-aggregation, it facilitated the formation of donor levels (E_D_), thus, Fermi level could shift toward the conduction band (E_C_) and the enhanced electron transport ability of LS·HCl-2 helical nanofibers was achieved. The electrical conductivity of LS·HCl-2 is ∼0.127 S/cm, higher than those of the reported aniline tetramers (AT; Figure S29) and PANI (Figure 3C). Hall effect experiments were performed at room temperature. Figure S30 displays the Hall effect measurement results on LS·HCl-2 and PANI samples at 298 K. The Hall resistance is linearly correlated with the magnetic field. From the slope of the linear correlation, the Hall coefficient is extracted to be −1.36 × 10^3^ cm^3^ C^−1^ in LS·HCl-2 and 0.042 cm^3^ C^−1^ in PANI. Hall effect indicates Hall mobilities in LS·HCl-2 is 172.72 cm^2^ V^−1^ S^−1^, which is larger than that in PANI (0.0053 cm^2^ V^−1^ S^−1^).

**Figure 4:**
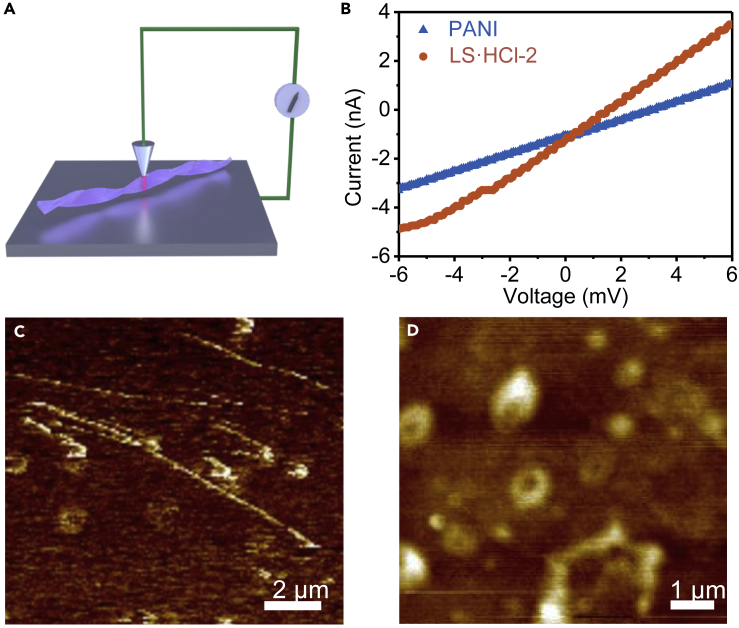
Conductivity estimation of the LP nanofiber and PANI nanoparticle from CAFM analysis (A) Schematic of the CAFM measurement setup for LS·HCl-2 nanofibers probing current. The red line represents the assuming preferred carrier transport pathway of carriers from the CAFM tip to the bottom Si wafer. (B–D) Typical I-V curves obtained for LS·HCl-2 nanofibers and PANI nanoparticles. (C and D) Typical AFM topographic image of (C) LS·HCl-2 and (D) PANI on Si wafer.

Conventional pressed-pellet measurements only measure the lower boundary of conductivity for nanostructures because the high contact resistance at the numerous junctions causes the overall conductivity to appear lower than the intrinsic conductivity of a single nanostructure.^43^ Using the measurement geometry depicted in Figures 4A and S32, electrical transport properties of the single LS·HCl-2 nanofiber, the LS-1 nanofiber and the PANI nanoparticle can be measured using conductive atomic force microscopy (CAFM), respectively. Typically, the conductivity of a single nanofiber of LS·HCl-2 calculated from I-V curves is ∼1.603 S/cm, which is 4.4 times higher than that of a single PANI nanosheet (0.362 S/cm) and 7781 times higher than an LS-2 nanofiber (Figures S31–S33), and rivals that of conventional unprocessed PANI, dedicating the efficient charge-carrier transmission within HCl doped AD J-aggregation structures.

### Electrochemical study of L-phenylalanine assemblies and polyanilines as well as stability analysis

**Figure 5:**
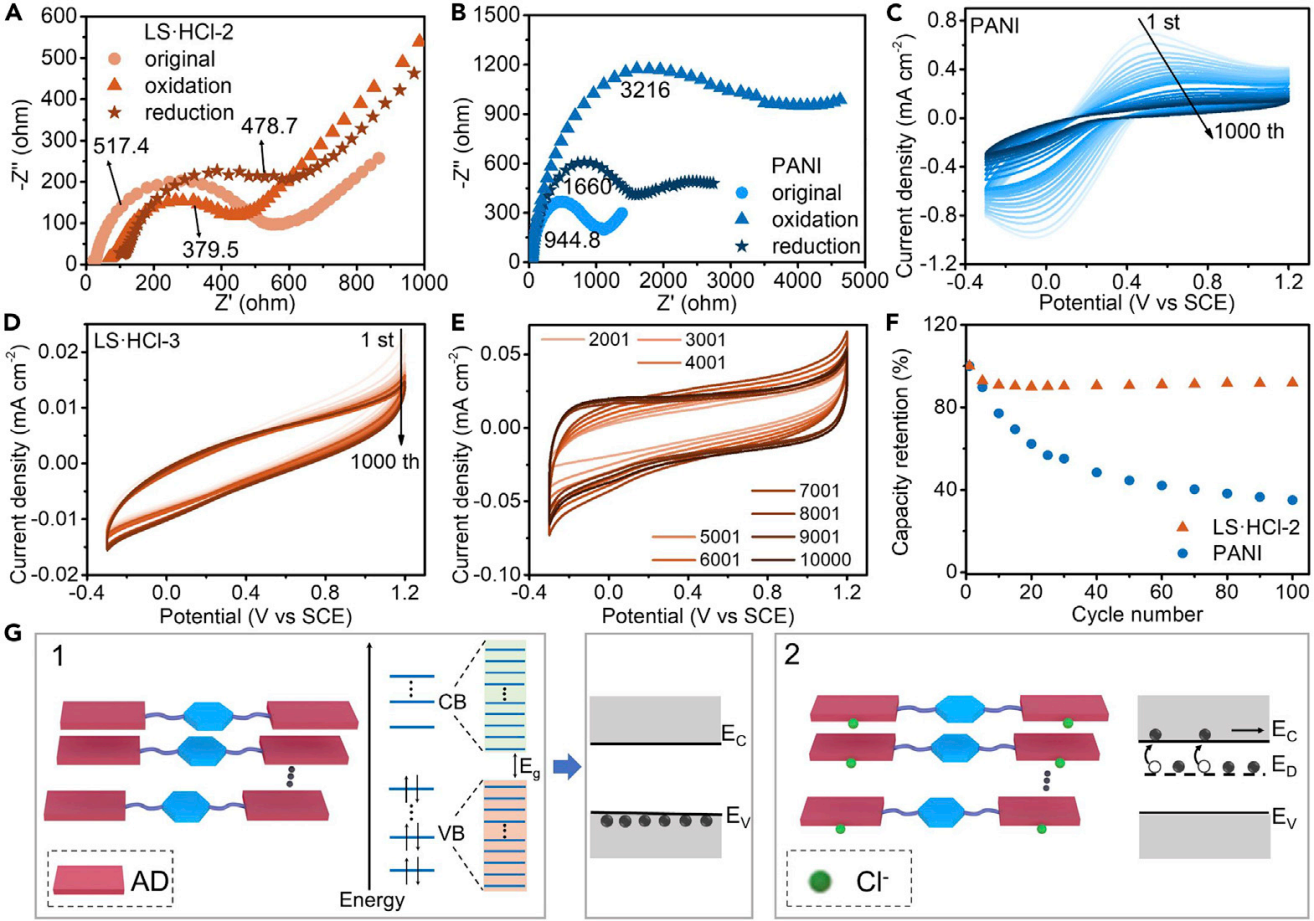
The stability and schematic diagram of conduction mechanisms of LS·HCl-2 (A–F) EIS of LS·HCl-2. Original: LS·HCl-2; Oxidation: the original LS·HCl-2 were oxidized by a constant applied voltage of 1.2 V for 600 s; Reduction: the oxidized LS·HCl-2 was reduced by a constant applied voltage of −0.3 V for 700 s (B) EIS of PANI. Original: HCl doped PANI was synthesized by emulsion polymerization; Oxidation: the original PANI was oxidized by a constant applied voltage of 1.2 V for 600 s; Reduction: the oxidized PANI was reduced by a constant applied voltage of −0.3 V for 700 s (C–F) The cyclic voltammograms of (C) PANI, (D and E) LS·HCl-2 at the scan rate of 500 mV/s, and a plot of (F) capacitance retention vs. cycle numbers. The number of cycles in (C and D) is 1, 5, 10, 15, 20, 25, 30, 40,50, 60, 70, 80, 90, 100, 200, 300, 400, 500, 600, 700, 800, 900, 1000. (G) Schematic diagram of the conduction mechanisms: 1, intermolecular hybridization of HOMO-LUMO levels; 2, Cl^−^ act as a donor dopant and donate electrons to AD segments after HCl doping to form E_D_.

The R_CT_ of LS·HCl-2 (571.8 Ω), LS·HCl-1 (1467 Ω), LS-1 (5191 Ω), and PANI (1261 Ω) were detected by electrochemical alternate impedance spectroscopy (EIS) (Table S1; Figure S34A). The R_CT_ (546.4 Ω) of DS·HCl-2 is close to that of LS·HCl-2. The R_CT_ of the mixed DP and LP assembly (mole ratio of 1:1) in 1-pentanol-HCl is about 2.3 times higher than those of LS·HCl-2 or DS·HCl-2 (Figure S34A; Table S1) because of the disrupted assembly structure of the coexisted molecules as proved by tadpole morphologies (Figure S14G). In addition, the variation of R_CT_ for LS·HCl-2 is less than 36.4% during oxidation or reduction (Figure 5A), while the variation is more than 240% for PANI (Figure 5B; Table S2). The photocurrent intensity of PANI decreases with decreasing potential from 1.2 to −0.3 V (Figure S35A) due to the decreased conductivity with reduction.^44^ While, the photocurrent intensities remain similar for LS·HCl-2 with reduction (Figure S35A; from 1.2 V to −0.3 V) and oxidation (Figure S35B; from −0.3 v to 1.2 V), indicating the stable conductive property of LS·HCl-2 in the potential ranges.

A cyclic voltammetry (CV) scan was conducted at different scan rates of 10, 20, 30, 40, 50, 60, 70, and 80 mV s^−1^ (Figures S36A and S36B) to investigate the nature of electrochemical reactions of LS·HCl-2 or PANI coated glassy carbon electrode (GCE). From CV, the maximum current is plotted vs the square root of the scan rate with a linear dependence on scan rate (Figures S36C and S36D), which is a typical response for an electroactive film.^45^ In contrast, a stronger linear relationship between the maximum current peak and the square root of the scan rates was shown for LS·HCl-2 than that of PANI. All cathodic and anodic scans of LS·HCl-2 overlapped, and the peaks were nearly symmetric. As the scan rate increased from 10 to 80 mV s ^−1^ (Figure S36A), the cathodic peaks at 0.051 V slightly shifted to a lower potential (by −89 mV for 0.051 V). In comparison, cyclic voltammograms of electrodes made from PANI exhibited a cathodic peak at 0.02V, moreover, the peak shifted by −149 mV as the scan rate increased (Figure S36B), indicating the redox process was less reversible than that of LS·HCl-2.

Cycling stability of LS·HCl-2 or PANI was investigated by monitoring the areal capacitance value from the CV scans. In total, 1000-10000 cycles at a voltage range of −0.3 V to 1.2 V were carried out at a scan rate of 500 mV s^−1^ (Figures 5C, 5D, 5E, and S37A). The areal capacitance value was calculated based on the following equation:

(Equation 1) $$Cs=\frac{S}{2\left(\Delta V\right)\cdot A\cdot k}$$

where Cs, S, ΔV, A, and k are the areal capacitance, area of the CV loop, potential window, electrode area (cm^2^), and scan rates (mV s ^−1^), respectively. Capacitance retention was calculated based on the following equation:

(Equation 2) $$\%capacitance=\frac{C_{n}}{C_{0}}\times 100\%$$

Where C_n_ is capacitance at n cycle and C_0_ is initial capacitance.

The plot of calculated capacitance and capacitance retention vs. cycle numbers is depicted in Figures 5F and S37B–S37D. The capacitance retention of the PANI sample was only 35.1% and 13.4% after 100 cycles and 1000 cycles, respectively. Conversely, LS·HCl-2 had obviously improved cycling stability with a capacitance retention of 91.9% and 120.6% after 100 cycles and 1000 cycles, respectively. Surprisingly, the current slightly increased with each cycle, indicating that LS·HCl-2 was becoming more and more electrochemically active (Figure 5E). In contrast, the current of PANI decreased with each cycle (Figures 5C and S37A), suggesting the degradation and excessive oxidation^46^ of PANI under an identical procedure, which was also confirmed by R_CT_ value and UV-Vis absorption spectrum obtained after 100, and 10,000 cycles. There is a huge increase in the system resistance of PANI after CV scanning because of an increase in the real-part impedance extrapolation of the low-frequency semicircle is (Figure S38A). In addition, the absorbance at the two characteristic peaks (around 350-420 nm and 530-710 nm) originating from the localized polarons and polaron band-π^∗^ band transitions^47^^,^^48^ was decreased in the UV-Vis absorption spectrum of PANI, which represents the reduction of conducting emeraldine salt (ES) phase of the polymer and further indicates an increase in the localized states in the polymer chains (Figure S38C). However, neither the R_CT_ value nor the UV-Vis absorption spectra of LS·HCl-2 showed considerable changes after 100 and 10,000 cycles, demonstrating the cycling stability of LS·HCl-2 (Figures S38B and S38D). Further, it was also found the differences between the NMR and XPS spectra of LS·HCl-2 and PANI, when after 2000 cycles. The intensity of the peaks in ^1^H NMR spectra of PANI at 6.53 and 5.72 ppm is much stronger than that of LS·HCl-2, indicating that PANI contained more degradation products (Figure S39). Meanwhile, there is a ∼1 eV shift of N 1s peak at 402.3 eV to higher energy region for PANI than that of LS·HCl-2 (Figures S40C and S40D), designating to higher protonation effects in PANI than in LS·HCl-2, thus PANI is more susceptible to nucleophilic attack than LS·HCl-2, then the electrochemical degradation of PANI is more likely to occur than LS·HCl-2.

### Conduction mechanism of L-phenylalanine assemblies

From the above results, the possible conduction mechanism for LS·HCl-2 can be speculated: Firstly, the highly regular packing-induced J-aggregation of AD segments enables the overlap of molecular orbits between neighbor molecules and in turn the intermolecular hybridization of HOMO-LUMO levels, which can create the low band-gap in the ordered helical nanofibers (Figures 5G-1). Secondly, Cl^−^ may donate electrons to AD units after HCl doping. Benefit from the well-ordered J-aggregation, E_D_ is formed and the electrons in E_D_ can be excited to the conduction band (E_C_) (Figures 5G-2), leading to macroscopic conduction of the nanofibers. The process for the formation of E_D_ can be demonstrated by time-resolved fluorescence and steady-state fluorescence techniques (Figure S41). Typically, the determinately separate oxidization or reduction units in LS·HCl-2 and intermolecular charge-carrier transmission in the materials may minimize redox influence on the conductivity, enhancing the stability of electrical properties during the redox process.

### L-phenylalanine assemblies act as a stable conductive layer and sensing layer

**Figure 6:**
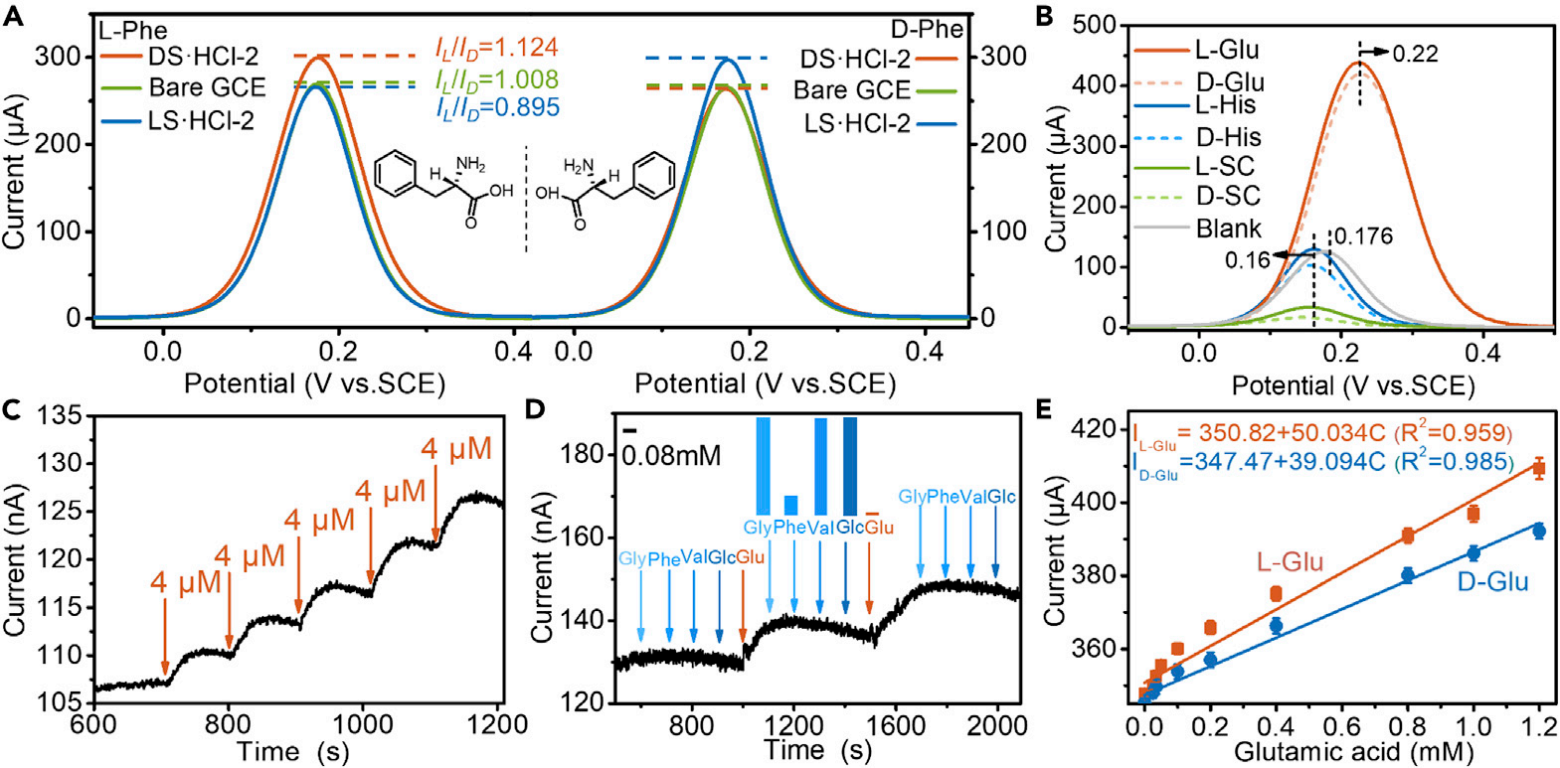
The sensors based on DS·HCl-2 or LS·HCl-2 (A) DPVs of residual L-Phe (left; DPV peak current of L-Phe denoted as IL) and D-Phe (right; DPV peak current of D-Phe denoted as ID) at the concentration of 5 mM in deionized water by GCE, DS·HCl-2 modified GCE, and LS·HCl-2 modified GCE. (B) DPVs of residual enantiomers glutamate (Glu), histidine (His), and sodium camphorsulfonate (SC) at the concentration of 5 mM in deionized water by DS·HCl-2 modified GCE. (Three electrodes; scan rate 50 mV/s; electrolyte: 0.2 M KCl and 5 mM K_3_[Fe(CN)_6_] in deionized water). (C) Amperometric responses of DS·HCl-2 modified GCE to successive addition of L-Glu at a constant potential of 0 V. (D) The selective sensing to Glu on DS·HCl-2 modified GCE. The concentrations are 0.08 mM for L-Glu, 0.4 mM for L-Phe, 2 mM for L-glycine (L-Gly), L-valine (L-Val) and glucose (Glc). The interfering compounds are part of the major elements that affect the detection of L-Glu in blood. (E) Linear calibration of the response current to the added L-Glu and D-Glu concentration on DS·HCl-2 modified GCE. The calibration of current response against L-Glu and D-Glu concentrations from 0.025 mM to 1.2 mM. Error bars for L-Glu or D-Glu concentration represent the SD from at least three independent measurements. The sensitivity of DS·HCl-2 modified GCE is calculated as high as 254.95 μA cm^−^^2^ mM^−^^1^ for L-Glu and 199.21 μA cm^−^^2^ mM^−^^1^ for D-Glu.

Taking the merit of the efficient and stable conductivity of LP and DP assemblies, the selective recognition sensors were constructed by coating one layer of LS·HCl-2 or DS·HCl-2 on GCE (Figure S42). By differential pulse voltammograms (DPV), a higher peak current for L-phenylalanine (L-Phe) than that for D-phenylalanine (D-Phe) was obtained on DS·HCl-2 modified GCE (Figure 6A). Typically, the sensor is quite stable and can be reused after at least 9 weeks of storage at room temperature (Figure S43A), while electrochemical sensors based on PANI are stable for less than 5 weeks (Table S6). Enantiomers of glutamate (Glu), histidine (His), serine methyl ester hydrochloride, and sodium camphorsulfonate (SC) can be sensitively detected on DS·HCl-2 modified GCE (Figures 6B, S44B, and S44D). DPV anodic peak potential of His shows a negative shift and positive shift for Glu compared with blank (Figures 6B, S44E, and S44F). The peak potential shift reflects the sensitivity of DP to the H^+^ concentration in the solution, which is a similar function to PANI.^49^^,^^50^ The sensor can specifically recognize Glu and the sensitivity may reach 254.95 μA cm^−2^ for 1 mM Glu on DS·HCl-2 conductive layer, which is about 2-50 times higher than the reported value^51^ (Figures 6C–6E; Table S7). Here, DS·HCl-2 or LS·HCl-2 can not only act as a conductive layer but also sensing layer.

### Conclusion

AD-derived nanofibrous materials with separate oxidization or reduction AD units during redox are prepared and show much stable electrical properties. The efficient self-assembly property of the nanofibers ensures the formation of J-aggregation of AD units, further inducing low band-gap formation. Typically, AD units can serve as effective transfer units to facilitate intermolecular charge-carrier transmission in the nanofibers. With doping, Cl^−^ can donate electrons to AD units to form E_D_ and create free electrons for the E_C_, ensuring the stable and efficient conduction of materials. These findings may overcome the shortcoming of the unstable electrical properties of PANI induced by the indeterminacy of oxidation sites and make it possible for cheap, easily available, low cost, and good soluble AD to be employed as conductive materials or electroactive moieties in a wide range of fields. Typically, the stability of electrical properties of conducting polymers (ICPs) is extremely susceptible to their oxidation state in chains as those of PANI. Thus, the method proposed in this study may also open an avenue to improve the stability of potentially other ICPs and enhance the electrical properties of conjugated organic small molecules.

### Limitations of the study

A facile synthesis of AD-derived nanofibers with stable and efficient electrical properties is described in the present study. However, it would be pertinent to investigate the electrical properties of oligomers of other conductive polymers, namely polypyrrole, poly(thiophene), poly (phenylenevinylene), and so forth, with a *C*_2_-symmetric structure, before generalizing the preparation method.

## STAR★Methods

### Key resources table

**Table:** 

REAGENT or RESOURCE	SOURCE	IDENTIFIER
**Chemicals, peptides, and recombinant proteins**		

N-(tert-butoxycarbonyl)-L-phenylalanine	Adamas	CAS: 13734-34-4
N-(tert-butoxycarbonyl)-D-phenylalanine	Adamas	CAS: 18942-49-9
1,4-benzenedicarbonyl dichloride	Adamas	CAS: 100-20-9
N-phenyl-1,4-phenylenediamine	Adamas	CAS: 101-54-2
1-hydroxybenzotriazole (HOBT)	Adamas	CAS: 2592-95-2
trifluoroacetic acid	Adamas	CAS: 76-05-1
N, N-diisopropylethylamine (DIEA)	Aladdin Chemicals	CAS: 7087-68-5
1,1,1,3,3,3-Hexafluoro-2-propanol	Aladdin Chemicals	CAS: 920-66-1
triethylamine	Aladdin Chemicals	CAS: 121-44-8
tetrabutylammonium hexafluorophosphate (Bu_4_NPF_6_)	Adamas	CAS: 3109-63-5
1-ethyl-3-(3-dimethylaminopropyl) carbodiimide hydrochloride (EDC-HCl)	Aladdin Chemicals	CAS: 25952-53-8
Hydrochloric acid (HCl)	Sinopharm Chemical Reagent Co., Ltd	CAS: 7647-01-0
dichloromethane (DCM)	Sinopharm Chemical Reagent Co., Ltd	CAS: 75-09-2
ethyl acetate	Sinopharm Chemical Reagent Co., Ltd	CAS: 141-78-6
ethanol	Sinopharm Chemical Reagent Co., Ltd	CAS: 64-17-5

**Software and algorithms**		

MDI JADE	Materials Data JADE	https://materialsdata.com/prodjd.html
Gatan Digital Micrograph	Gatan, Inc.	https://www.gatan.com/products/tem-analysis/digitalmicrograph-software
MestReNova	Mestrelab Research	https://mestrelab.com/
Gaussian 09	Frish et al.	https://Gaussian.com
OriginPro 9.0	OriginLab	https://www.originlab.com/
Chem3D	PerkinElmer	https://www.3dchem.com/

**Other**		

FTIR spectrometer	ThermoFisher Scientific	N/A
X-ray diffraction spectrometer	Bruker	N/A
Quantum design physical property measurement system (PPMS-9T)	Quantum Design	N/A
CD spectrometer	JASCO	N/A
X-ray photoelectron spectroscopy	Kratos Analytical	N/A
Transmission electron microscopic (TEM) and selected area electron diffraction (SAED)	ThermoFisher Scientific	N/A
Diffuse reflectance ultraviolet-visible-near-infrared (UV-Vis-NIR) spectroscopy	PerkinElmer	N/A
UV-vis spectra	ThermoFisher Scientific	N/A
Scanning electron microscopy (SEM)	FEI	N/A
Specific surface area	Micromeritics Instrument Corp	N/A
NMR spectra	Bruker	N/A
High-resolution mass spectra (HRMS)	Bruker	N/A

### Resource availability

#### Lead contact

Further information and requests for resources should be directed to and will be fulfilled by the lead contact, C.F. (clfeng@sjtu.edu.cn).

#### Materials availability

All materials generated in this study are available from the lead contact without restriction.

### Experimental model and subject details

This work does not use experimental models typical in the life sciences.

### Method details

#### Samples preparation

##### L/DP

N-(tert-butoxycarbonyl)-L-phenylalanine (5.31 g, 20.00 mmol) and HOBT (3.51g, 26 mmol) was added to dry DCM containing N-phenyl-1,4-phenylenediamine (3.68g, 20 mmol) and DIEA (86 mmol). After the reaction mixture was stirred at 0 °C for 0.5 h, the EDC-HCl (40 mmol) in dry DCM was added dropwise to this solution. All the solvents were evaporated under vacuum and the residue was subsequently dissolved in ethyl acetate. After recrystallization, the undissolved substance was collected and dried to give dark purple crystals (Boc-L-PhAD 6.94 g, 80.2%). Compound Boc-L-PhAD (6.94 g, 16.04 mmol) was treated with trifluoroacetic acid (23 mL) in DCM (40 mL) for 2 h. After evaporation under vacuum conditions the residue was pumped to dryness. The residue was dissolved in DCM (60 mL) and triethylamine (16.0 mL) was added. To this solution was added 1,4-benzenedicarbonyl dichloride (1.63 g, 8 mmol) in batches, then stirred at room temperature for 12 h and gel-like precipitate formed. The gel phase was filtered, washed with deionized water, and finally dried in the vacuum oven to give LP (5.6 g, 7.06 mmol, 88.25%). Similarly, DP was obtained as a white solid (5.1g, 80.4%).

##### L/D-PheAn

N-(tert-butoxycarbonyl)-L-phenylalanine (5.31 g, 20.00 mmol) and HOBT (3.51 g, 26 mmol) was added to dry dichloromethane (DCM) containing aniline (1.86 g, 20 mmol) and DIEA (86 mmol). After the reaction mixture was stirred at 0 °C for 0.5 h, the EDC-HCl (40 mmol) in dry dichloromethane was added dropwise to this solution. All the solvents were evaporated under vacuum and the residue was subsequently dissolved in ethyl acetate. After recrystallization, the undissolved substance was collected and dried to give white crystals (Boc-L-PhAn 5.06 g, 74.4%). Compound Boc-L-PhAn (5.06 g, 14.87 mmol) was treated with trifluoroacetic acid (16.5 mL) in DCM (40 mL) for 2 h. After evaporation under vacuum conditions the residue was pumped to dryness. The residue was dissolved in DCM (70 mL) and triethylamine (15 mL) was added. To this solution was added 1,4-benzenedicarbonyl dichloride (1.51 g, 7.43 mmol) in batches, then stirred at room temperature for 12 h and gel-like precipitate formed. The gel phase was filtered, washed with deionized water, and finally dried in the vacuum oven to give L-PheAn (4.9 g, 7.06 mmol, 80.3%). Similarly, D-PheAn was obtained as a white solid (5.3 g, 86.9%).

##### PANI

Synthesis of PANI was carried in acidic media with 0.1M HCl. Solution A: Aniline monomer purified by distillation (0.97 g, 10 mmol) and SDS(SDS; 1.5 g, 5 mmol) was added to 0.1M HCl (30 mL). Solution B: Ammonium persulfate (APS; 2.377g, 10 mmol) was dissolved in 0.1M HCl (30 mL). Solution A added drop by drop into the solution B under constant stirring, and agitation speed was controlled at 300 rpm for 6 h. The product of polymerization reaction was then filtered from the solution and subsequently washed with acetone and ethanol for several times until the pH of the solution reached 6. Then the product was filtered again, and finally dried in the vacuum oven to give PANI (0.71g).

L-2Phe was synthesized according to procedures described in the literature.^52^

#### Measurement of band gap

##### Electrochemical gaps

All the electrochemical experiments were conducted with a CHI660E electrochemistry workstation (Shanghai Chenhua Instruments Co., China). Electrochemical gaps were determined from the cyclic voltammetry (CV) experiments which were employed in dehydrated and deoxygenated dichloromethane (0.1 M Bu_4_NPF_6_ as the supporting electrolyte). A platinum foil (1 × 5 mm) was used as the auxiliary electrode and an Ag/AgCl electrode as the reference electrode. The LP/GCE (10 μL solution containing LP assemblies drop-cast onto GCE with a diameter of 5 mm) or PANI/GCE was used as the working electrode. Then the CV scan potential sweep range of −1.8 V–1.8 V, the scanning speed of 50 mV/s, and Eox,onset and Ered, onset determined from the onset potentials of the oxidation and reduction waves respectively. All cyclic voltammograms were calibrated against Fc/Fc^+^ redox couple in respective solvents. The electrochemical gap is calculated from the difference of oxidation and reduction potentials, and the calculation formula is as follows:

$$E_{HOMO}^{elec}=-\left(E_{ox,onset}+4.8\right)eV;\;E_{LUMO}^{elec}=-\left(E_{red,onset}+4.8\right)eV$$

##### Optical gaps

The optical gap can be determined by the Kubelka–Munk function in the range of diffuse reflectance spectra. For the semiconductor, the correlation between photon energy (*hν*) and the coefficient of absorption (A) can be expressed as^53^^,^^54^

$${\left(Ahv/K\right)}^{n}=hv-E_{g}$$

where K is the absorption constant for direct transitions, A is the diffuse reflectance UV absorbance, *hν* = 1240/λ, and *n* is an index which depends on the nature of the electronic transition responsible for the absorption. For direct band gap n = ½ and indirect band gap n = 2,^55^ In organic semiconductors one usually assumes n = ½.^56^

##### DFT approach

Geometrical and HOMO/LUMO analyses by using DFT/M06-2X, 6-311+G∗∗Methods.

#### Measurement of electrical performance

##### Pressed-pellet conductivity

After milling the samples (LP assemblies LS·HCl-2, PANI), 200 mg of each sample was pressed into tablets by a tablet press. The conductivities of the samples were tested at 300 K on a quantum design physical property measurement system (PPMS-9T) instrument using a four-probe configuration.

##### Hall effect experiment

After milling the samples (LS·HCl-2, PANI), 200 mg of each sample was pressed into tablets by a tablet press. The Hall resistance of the samples were carried out with direct current, under the magnetic field from -1T to 1T at 300K on PPMS-9T.

##### Conductive AFM

Conductive AFM measurements were carried out for LS·HCl-2 and PANI with vertical orientations with respect to Si wafer, the test method was described in the literature.^57^ Transport properties in vertical orientations is measured when a metallic AFM tip serves as the top electrode contact to the apex of a nanoparticle, in combination with the Si substrate as the bottom electrode.

##### Electrochemical impedance spectroscopy

A platinum foil (1 × 5 mm) was used as the auxiliary electrode and a saturated calomel electrode (SCE) electrode as the reference electrode. Prepared L- or DP assemblies/glass (the size of glass electrode is 6 × 10 mm) electrode was used as the working electrode (the copper wire as a conductor). Electrochemical impedance spectroscopy (EIS) was employed in 5.0 mM K_3_Fe(CN)_6_ - K_4_Fe(CN) _6_ and 0.1 M KCl solution at open potential.

##### Photoresponse test

Photoresponse of L/DP assemblies and PANI were carried out with a PLS-SXE300/300 UV xenon lamp system, wherein a set of long pass filters were applied, at bias voltages between 1 and −1 V under 365 nm, 400-780 nm, 800-1000 nm radiation.

##### Mott-Schottky (M−S) measurement

A three-electrode configuration with the LP assemblies/glass (or PANI/glass) as working electrode, standard calomel electrode (SCE) as reference electrode and platinum electrode as counter electrode is used for measurement. KCl (0.2 M) is used as electrolyte. All the measurements are done under dark conditions.

#### Performance measurement of electrochemical sensor

The preparation process of electrochemical sensor is as follows: A 2 mg D/LP powers (LS-1, LS·HCl-1, LS·HCl-2, etc. in dried form) were dispersed in a 50 μL mixture of water and acetic acid (9:1, v/v) containing 1 mg/mL chitosan for ultrasonic agitation for 5 min. Then, D/LP mixture was drop cast (10 μL) using a micropipette on cleaned glassy carbon electrode (GCE) and dried for 12 h at room temperature. A platinum foil (1 × 5 mm) was used as the auxiliary electrode and a saturated calomel electrode (SCE) electrode as the reference electrode. L/DP/GCE electrochemical sensor was used as the working electrode.

##### Different potential voltammograms (DPV)

DPV was employed in 5.0 mM K_3_Fe(CN)_6_ (served as an electrochemical probe) and 0.2 M KCl solution at scan rate of 50 mV/s.

##### Cyclic voltammetry (CV)

CV was employed in 5.0 mM K_3_Fe(CN)_6_ and 0.2 M KCl solution at scan rate of 50 mV/s.

##### Chronoamperometry

The chronoamperometry was carried out in 0.2 M KCl solution at constant potential of 0 V.

#### Structural characterization

##### Circular dichroism (CD) spectra

CD and dynamic CD spectra of L/DP assemblies were recorded in the UV region (190-400 nm) using a 0.1 mm quartz cuvette at a concentration of 3 mg/mL using a JASCO J-815 spectrometer.

##### Diffuse reflectance ultraviolet-visible-near-infrared (UV-Vis-NIR) spectroscopy

UV–VIS–NIR spectrophotometer model (Lamda 950) in the wavelength range from 200 to 2200 nm was employed to determine the diffused reflectance (DR) measurements. The system was designed by using the integrating sphere attachment with BaSO_4_ as a reference material.

##### UV-vis spectra

UV-vis spectra were obtained using a Thermo Fisher UV/EV300 spectrophotometer.

##### Fluorescence emission spectra

Fluorescence spectra recorded on steady-state and time-resolved fluorescence spectrofluorometer (QM/TM/IM).

##### Fourier transform infrared (FTIR) spectra

FTIR spectra of the samples were acquired using a ThermoFisher Scientific FT-IR Instrument. The KBr disk technique was used for the solid-state measurements. The samples were scanned between 4000 and 400 cm^−1^ wavelengths at an interval of 1.9285 cm^−1^.

##### Scanning electron microscopy (SEM)

The samples were prepared by applying drops of the diluted suspension (at a concentration of 3 mg/mL) onto silicon wafers, followed by drying and coating them with a thin layer of Au to increase the contrast, which were measured by SEM (Sirion 200 & INCA X-Act Microscope).

##### Transmission electron microscopic (TEM) and selected area electron diffraction (SAED)

LP assemblies were studied using a Talos F200X G2 TEM operated at 200 keV. The samples for TEM and SAED were prepared by applying drops of LP assemblies suspension onto copper grids, which were then dried under ambient conditions before measuring. SAED patterns were collected on multiple areas on each crystal, which showed identical patterns that confirmed their single crystalline nature. Multiple crystals were analyzed for each sample and reproducible results were obtained.

##### X-ray diffraction (XRD)

Powder XRD analysis was conducted using XRD equipment (D8 Advance, Bruker) with Cu Kα radiation.

##### X-ray photoelectron spectroscopy (XPS)

The XPS results of the LP assemblies were collected using AXIS Ultra DLD photoelectron spectrometer (Kratos Analytical).

##### Specific surface area

Specific surface area characterization of DP assemblies was evaluated using an accelerated surface area and porosimetry system (ASAP 2460).

##### NMR spectra

NMR spectra were recorded on a Bruker AVANCE III HD 500 Instrument (500 MHz) spectrometer at 298 K using partially deuterated solvents as internal standards. Coupling constants (J) are denoted in Hz and chemical shifts (*δ*) in ppm. Multiplicities are denoted as follows: s = singlet, d = doublet, t = triplet, m = multiplet. See the supporting information for the NMR spectra of all new compounds.

##### High-resolution mass spectra (HRMS)

HRMS were recorded in the positive ion mode with a Buker impact II mass spectrometer. See the supporting information for the HRMS spectra of all new compounds.

### Quantification and statistical analysis

Figures were produced by Origin from the raw data. Error bars represent the SEM.

## Data Availability

• All data reported in this article are available within the paper and the supplementary information files. • This article does not report original code. • Any additional information required to reanalyze the data reported in this paper is available from the lead contact upon reasonable request.
