# Supplementary material for: Aniline dimers serving as stable and efficient transfer units for intermolecular charge-carrier transmission
Source: iScience. 2022 Dec 8;26(1):105762. doi: 10.1016/j.isci.2022.105762 (PMC9804111; doi:10.1016/j.isci.2022.105762)
Supplement: Document S1. Figures S1–S48 and Tables S1–S8 [file mmc1.pdf]

iScience, Volume *26*

Supplemental information

Aniline dimers serving as stable and efficient transfer units for intermolecular charge-carrier transmission

Juexin Huang and Chuanliang Feng

Supplemental information

Aniline Dimers Serving as Stable and Efficient Transfer Units for Intermolecular Charge-Carrier Transmission

Juexin Huang[1], and Chuanliang Feng[1, 2, *]

[1]State Key Lab of Metal Matrix Composites, School of Materials Science and Engineering, Shanghai Jiao Tong University, 800 Dongchuan Road, Shanghai 200240, P. R. China.

[2]Lead Contact

*Correspondence: clfeng@sjtu.edu.cn

Contents:

I. Supplemental Figures

1.1. Oxidation process in PANI and LP molecules (S1)
1.2. Intermolecular arrangements and narrow band-gap of LP assemblies (S2-S25)
1.3. Effect of HCl doping on the energy band structure of LP assemblies (S26-S33)
1.4. Electrochemical study of LP assemblies and PANI as well as stability analysis (S34-S41)
1.5. LP assemblies act as stable conductive layer and sensing layer (S42-S46)
1.6. Infrared spectra and UV-vis spectra of PANI (S47-S48)

II. Supplemental Tables

I. Supplemental Figures

1.1. Oxidation process in PANI and LP molecules

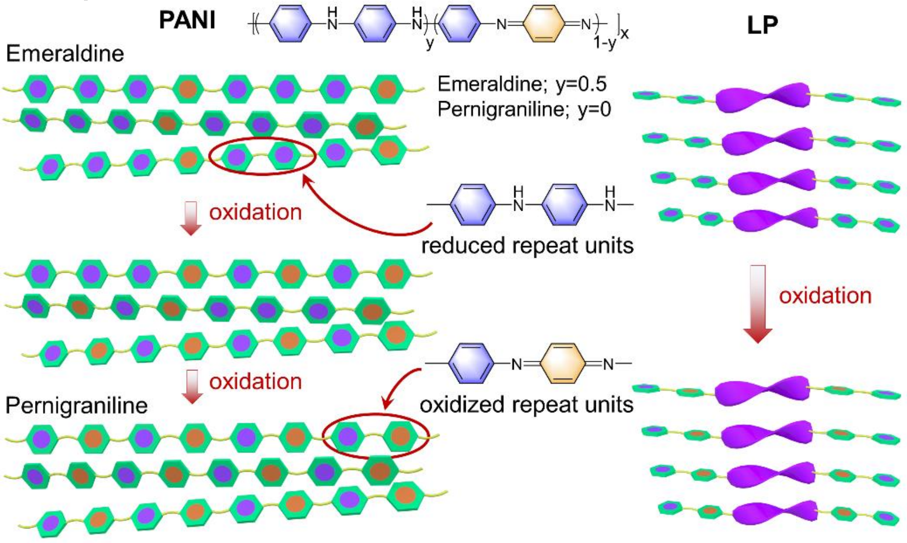

Figure S1. Chain structure of PANI and the oxidation process in PANI and LP molecules. Related to Figure 1.

1.2. Intermolecular arrangements and narrow band-gap of LP assemblies

^{1}H, ^{13}C NMR and HRMS spectra

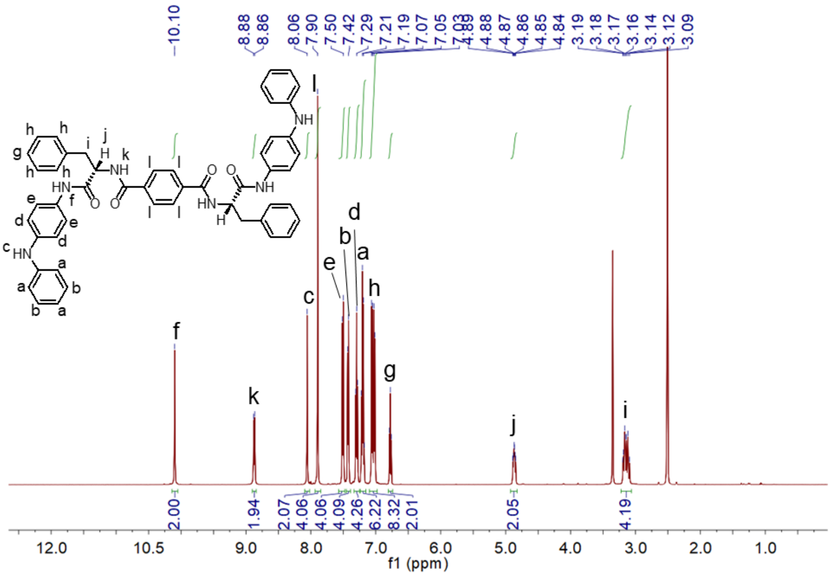

Figure S2. The ^{1}H NMR spectra of LP in DMSO-*d6*. Related to STAR Methods.

^{1}H NMR (500 MHz, DMSO) δ 10.10 (s, 2H), 8.87 (d, J = 8.1 Hz, 2H, NH), 8.06 (s, 2H, Ar-H), 7.90 (s, 4H, Ar-H), 7.51 (d, J = 8.8 Hz, 4H, Ar-H), 7.43 (d, J = 7.4 Hz, 4H, Ar-H), 7.29 (t, J = 7.6 Hz, 4H, Ar-H), 7.22-7.18 (m, 6H, Ar-H), 7.04 (dd, J = 18.2, 8.3 Hz, 8H, Ar-H), 6.78 (t, J = 7.3 Hz, 2H, CH), 4.87 (m, J = 9.7, 5.0 Hz, 2H), 3.19-3.09 (m, 4H, CH_2).

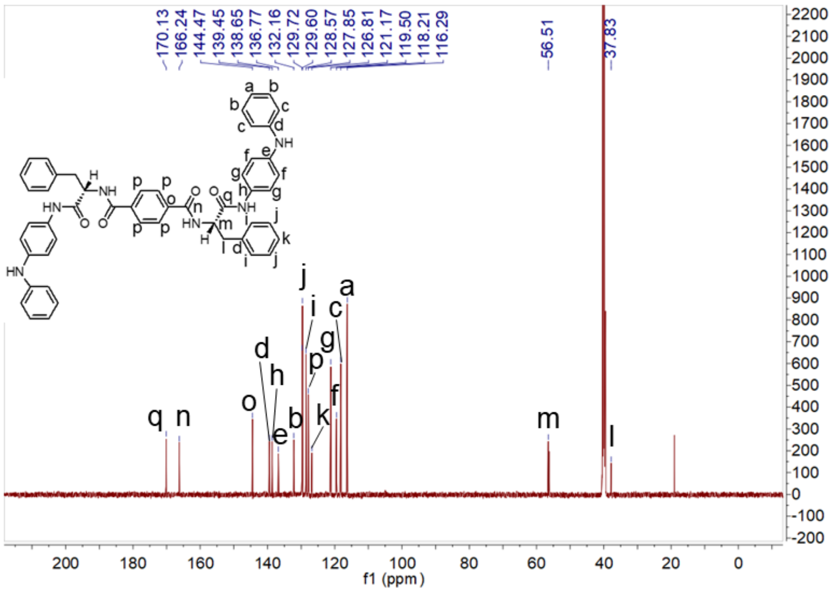

Figure S3. The ^{13}C NMR spectra of LP in DMSO-*d6*. Related to STAR Methods.
^{13}C NMR (126 MHz, DMSO) δ 170.13 (s), 166.24 (s), 144.47 (s), 139.45 (s), 138.65 (s), 136.77 (s), 132.16 (s), 129.66 (d, J = 14.1 Hz), 128.57 (s), 127.85 (s), 126.81 (s), 121.17 (s), 119.50 (s), 118.21 (s), 116.29 (s), 56.51 (s), 37.83 (s).

Mass Spectrum SmartFormula Report

Analysis Info

		Acquisition Date	11/27/2020 2:59:59 PM	
Analysis Name	D:\Data\GROUP\fengchuanliang\huangyuxin\3_1-3_01_2294.d			
Method	HPLC_wo column_MS_pos.m	Operator	Demo User	
Sample Name	3	Instrument	impact II	1825265.10257
Comment				

Acquisition Parameter

Source Type	ESI	Ion Polarity	Positive	Set Nebulizer	2.0 Bar
Focus	Not active	Set Capillary	4500 V	Set Dry Heater	220 °C
Scan Begin	50 m/z	Set End Plate Offset	-500 V	Set Dry Gas	8.0 l/min
Scan End	1300 m/z	Set Charging Voltage	2000 V	Set Divert Valve	Waste
		Set Corona	0 nA	Set APCI Heater	0 °C

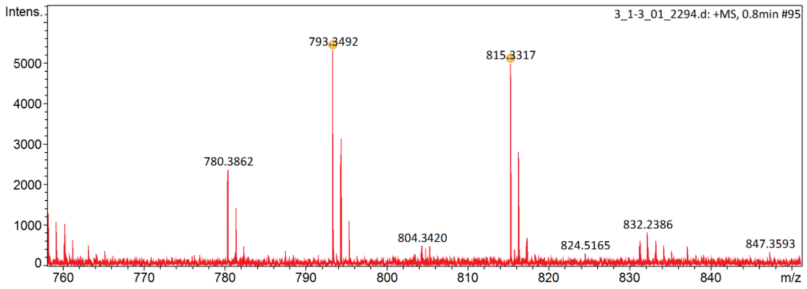

Meas. m/z	#	Ion Formula	m/z	Sum Formula	err [ppm]	mSigma	# mSigma	Score	rdb	e⁻ Conf	N-Rule
793.3492	1	C50H45N6O4	793.3497	C50H44N6O4	0.6	24.9	1	100.00	32.0	even	ok
815.3317	1	C50H44N6NaO4	815.3316		-0.1	19.0	1	100.00	32.0	even	ok

Figure S4. The HRMS spectra of LP in tetrahydrofuran. Related to STAR Methods.
HRMS (ESI) Calculated for $[C_{50}H_{45}N_6O_4]^+$, $([M+H]^+)$: 793.3497, found 793.3492. $[C_{50}H_{44}N_6O_4Na]^+$, $([M+Na]^+)$: 815.3316, found 815.3317.

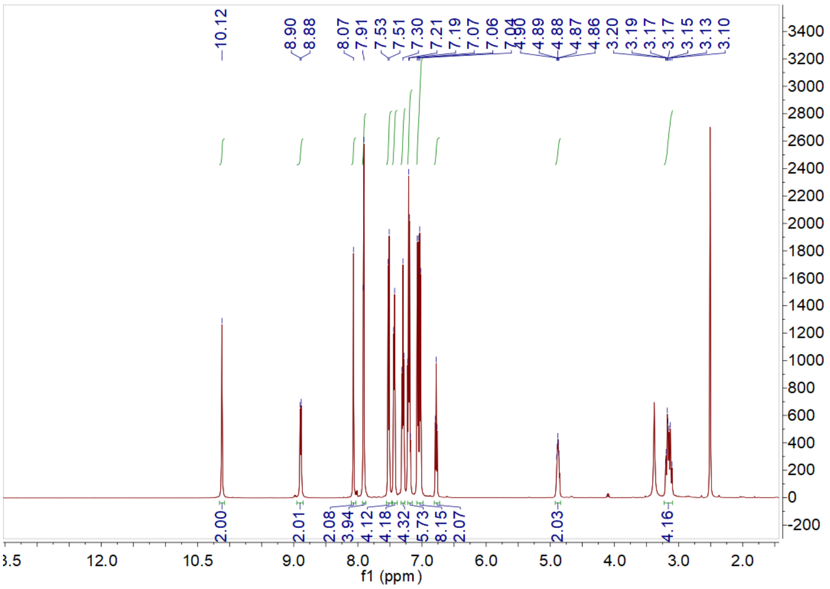

Figure S5. The ^{1}H NMR spectra of DP in DMSO-*d6*. Related to STAR Methods.

^{1}H NMR (500 MHz, DMSO) δ 10.12 (s, 2H), 8.89 (d, J = 8.1 Hz, 2H, NH), 8.05 (s, 2H, NH), 7.91 (s, 4H, Ar-H), 7.50 (d, J = 8.8 Hz, 4H, Ar-H), 7.43 (d, J = 7.4 Hz, 4H, Ar-H), 7.30 (t, J = 7.4 Hz, 4H, Ar-H), 7.22-7.18 (m, 6H, Ar-H), 7.05 (dd, J = 18.9, 8.3 Hz, 8H, Ar-H), 6.78 (t, J = 7.3 Hz, 2H, Ar-H), 4.88 (m, J = 10.0, 5.0 Hz, 2H), 3.20-3.10 (m, 4H, CH_2).

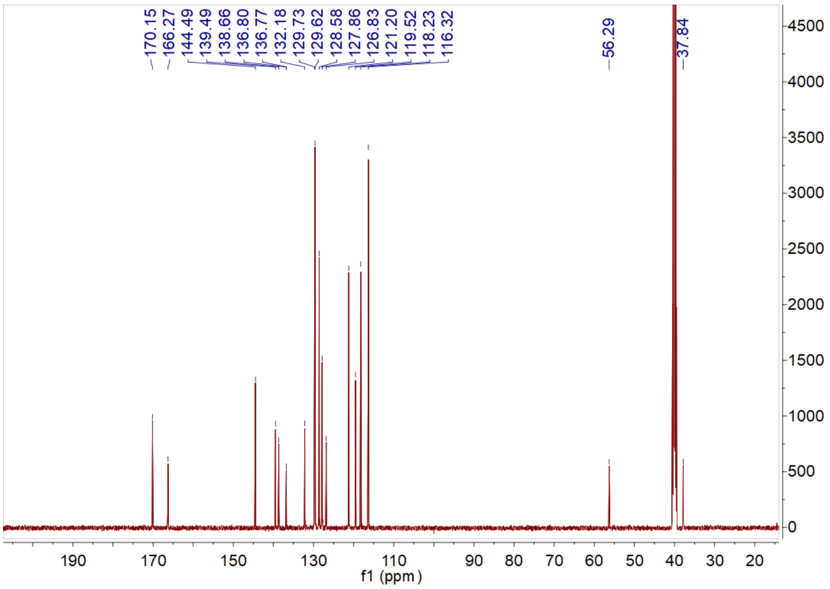

Figure S6. The ^{1}H NMR spectra of DP in DMSO-*d6*. Related to STAR Methods.

^{13}C NMR (126 MHz, DMSO) δ 170.15 (s), 166.27 (s = 6), 144.49 (s), 139.49 (s), 138.66 (s), 136.78 (d, J = 3.8 Hz), 132.18 (s), 129.68 (d, J = 14.1 Hz), 128.58 (s), 127.86 (s), 126.83 (s), 121.20 (s), 119.52 (s), 118.23 (s), 116.32 (s), 56.29 (s), 37.84 (s).

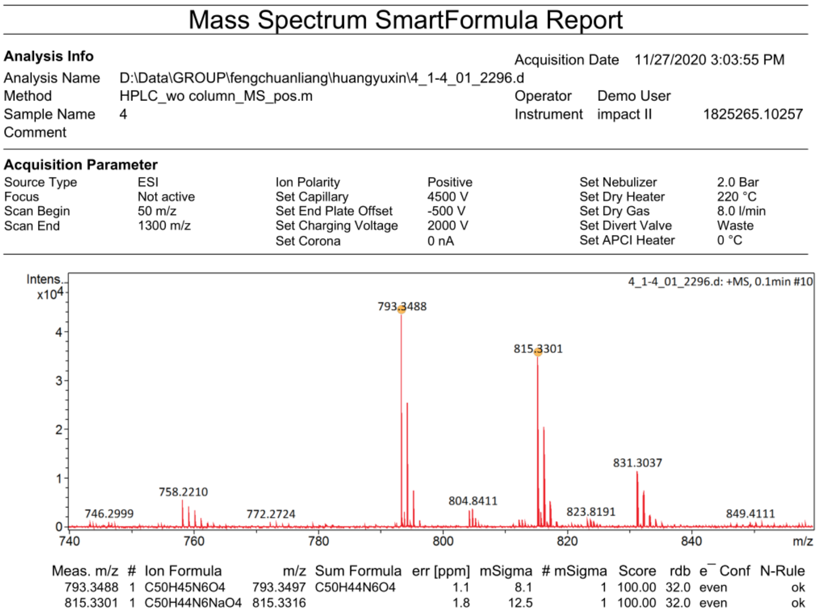

Mass Spectrum SmartFormula Report

Analysis Info

Analysis Name	D:\Data\GROUP\fengchuanliang\huangyuxin\4_1-4_01_2296.d	Acquisition Date	11/27/2020 3:03:55 PM
Method	HPLC_wo column_MS_pos.m	Operator	Demo User
Sample Name	4	Instrument	impact II 1825265.10257
Comment			

Acquisition Parameter

Source Type	ESI	Ion Polarity	Positive	Set Nebulizer	2.0 Bar
Focus	Not active	Set Capillary	4500 V	Set Dry Heater	220 °C
Scan Begin	50 m/z	Set End Plate Offset	-500 V	Set Dry Gas	8.0 l/min
Scan End	1300 m/z	Set Charging Voltage	2000 V	Set Divert Valve	Waste
		Set Corona	0 nA	Set APCI Heater	0 °C

Meas. m/z	#	Ion Formula	m/z	Sum Formula	err [ppm]	mSigma	# mSigma	Score	rdb	e⁻ Conf	N-Rule
793.3488	1	C50H45N6O4	793.3497	C50H44N6O4	1.1	8.1	1	100.00	32.0	even	ok
815.3301	1	C50H44N6NaO4	815.3316		1.8	12.5	1	100.00	32.0	even	ok

Figure S7. The HRMS spectra of DP in tetrahydrofuran. Related to STAR Methods.
HRMS (ESI) Calculated for $[C_{50}H_{45}N_6O_4]^+$, $([M+H]^+)$: 793.3497, found 793.3488. $[C_{50}H_{44}N_6O_4Na]^+$, $([M+Na]^+)$: 815.3316, found 815.3301.

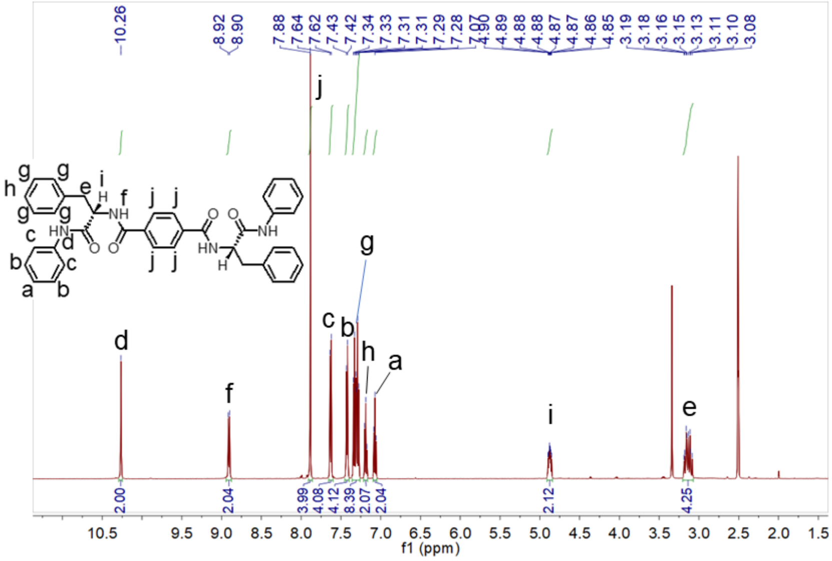

Figure S8. The ^{1}H NMR spectra of L-PheAn in DMSO-*d6*. Related to STAR Methods.
^{1}H NMR (500 MHz, DMSO-d6) δ 10.26 (s, 2H), 8.91 (d, J = 8.1 Hz, 2H), 7.88 (s, 4H, Ar-H), 7.63 (d, J = 7.6 Hz, 4H, Ar-H), 7.42 (d, J = 7.2 Hz, 4H, Ar-H), 7.31 (dt, J = 18.5, 7.7 Hz, 8H, Ar-H), 7.19 (t, J = 7.4 Hz, 2H, Ar-H), 7.07 (t, J = 7.4 Hz, 2H, Ar-H), 4.87 (m, J = 10.2, 8.3, 4.9 Hz, 2H, CH), 3.14 (m, J = 23.9, 13.7, 7.6 Hz, 4H, CH_2).

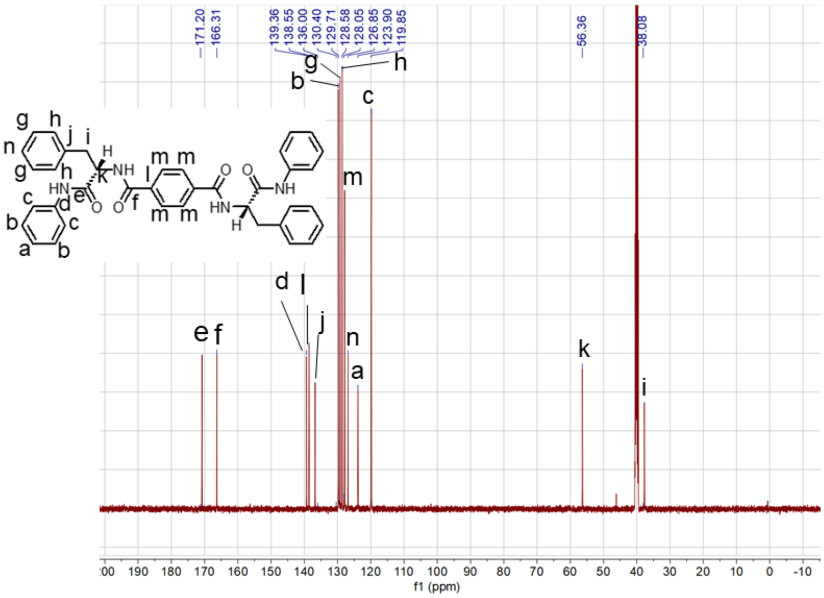

Figure S9. The ^{13}C NMR spectra of L-PheAn in DMSO-*d6*. Related to STAR Methods.
^{13}C NMR (126 MHz, DMSO) δ 170.11 – 170.02 (m), 166.24 (s), 144.47 – 144.37 (m), 139.43 – 139.33 (m), 138.64 (s), 136.77 – 136.67 (m), 132.16 (s), 129.71 (s), 129.60 (s), 128.56 (s), 127.84 (s), 126.81 (s), 121.19 (s), 119.50 (s), 118.22 (s), 116.31 (s), 56.26 (s), 37.83 (s).

Mass Spectrum SmartFormula Report

Analysis Info

Analysis Name	D:\Data\GROUP\fengchuanliang\huangyuxin\2_1-2_01_2292.d	Acquisition Date	11/27/2020 2:56:04 PM
Method	HPLC_wo column_MS_pos.m	Operator	Demo User
Sample Name	2	Instrument	impact II 1825265.10257
Comment			

Acquisition Parameter

Source Type	ESI	Ion Polarity	Positive	Set Nebulizer	2.0 Bar
Focus	Not active	Set Capillary	4500 V	Set Dry Heater	220 °C
Scan Begin	50 m/z	Set End Plate Offset	-500 V	Set Dry Gas	8.0 l/min
Scan End	1300 m/z	Set Charging Voltage	2000 V	Set Divert Valve	Waste
		Set Corona	0 nA	Set APCI Heater	0 °C

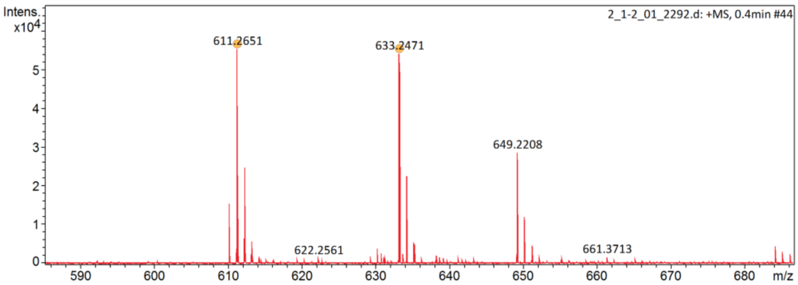

Meas. m/z	#	Ion Formula	m/z	Sum Formula	err [ppm]	mSigma	# mSigma	Score	rdb	e⁻ Conf	N-Rule
611.2651	1	C38H35N4O4	611.2653	C38H34N4O4	0.4	6.7	1	100.00	24.0	even	ok
633.2471	1	C38H34N4NaO4	633.2472		0.3	7.4	1	100.00	24.0	even	ok

Figure S10. The HRMS spectra of L-PheAn in tetrahydrofuran. Related to STAR Methods.
HRMS (ESI) Calculated for $[C_{38}H_{35}N_4O_4]^+$, $([M+H]^+)$: 611.2653, found 611.2651. $[C_{38}H_{34}N_4O_4Na]^+$, $([M+Na]^+)$: 633.2472, found 633.2471.

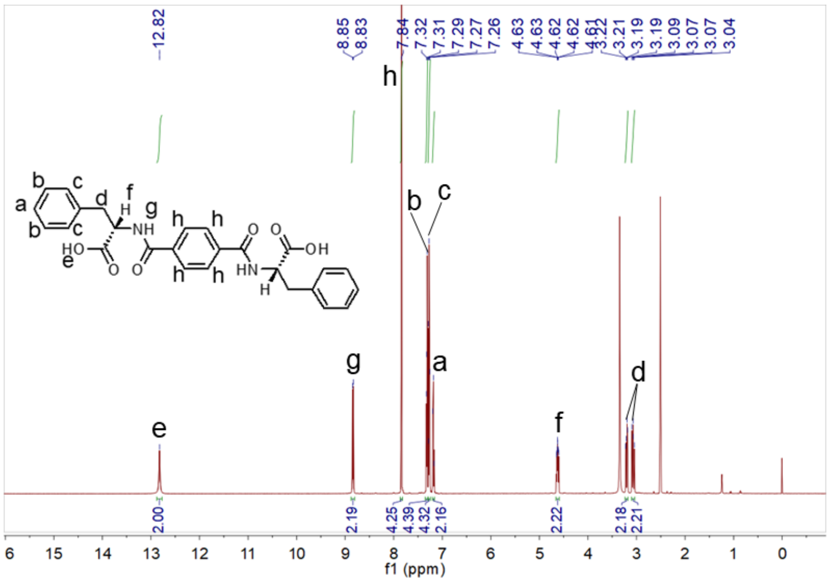

Figure S11. The ¹H NMR spectra of L-2Phe in DMSO-*d6*. Related to STAR Methods.
^{1}H NMR (500 MHz, DMSO-d6): δ3.04-3.22 (m, 4H, CH_2), 4.60-4.65 (m, 2H, CH), 7.18 (t, J = 6.6 Hz, 2H, Ar-H), 7.27 (dd, J = 10.3, 4.8 Hz, 4H, Ar-H) , 7.35-7.30 (m, 4H, Ar-H), 7.84 (s, 4H, Ar-H), 8.84 (d, J = 8.2 Hz, 2H, CO-NH), 12.82 (s, 2H, COOH).

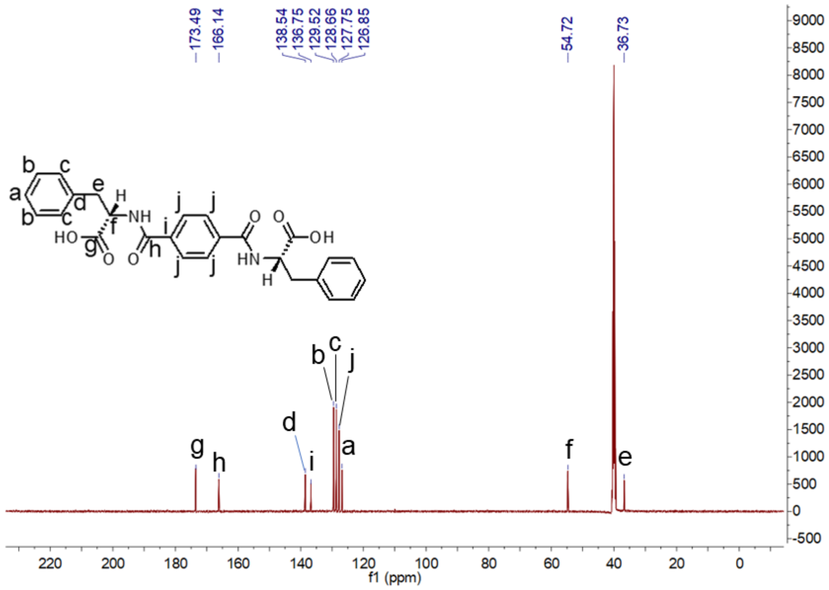

Figure S12. The ¹³C NMR spectra of L-2Phe in DMSO-*d6*. Related to STAR Methods.
^{13}C NMR (126 MHz, DMSO-d6) δ 173.49 (s), 166.14 (s), 138.54 (s), 136.75 (s), 129.52 (s), 128.66 (s), 127.75 (s), 126.85 (s), 54.72 (s), 36.73 (s).

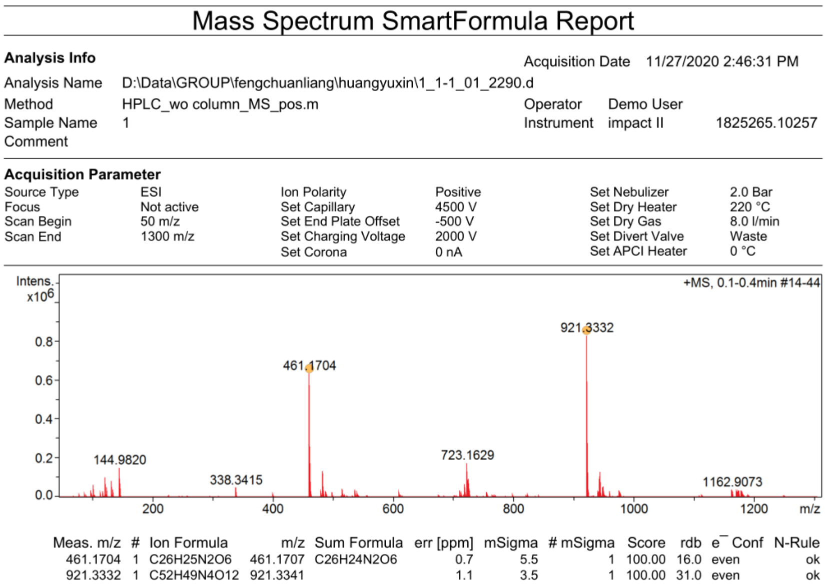

Figure S13. The HRMS spectra of L-2Phe in tetrahydrofuran. Related to STAR Methods. HRMS (ESI) Calculated for $[C_{26}H_{25}N_2O_6]^+$, $([M+H]^+)$: 461.1707, found 461.1704.

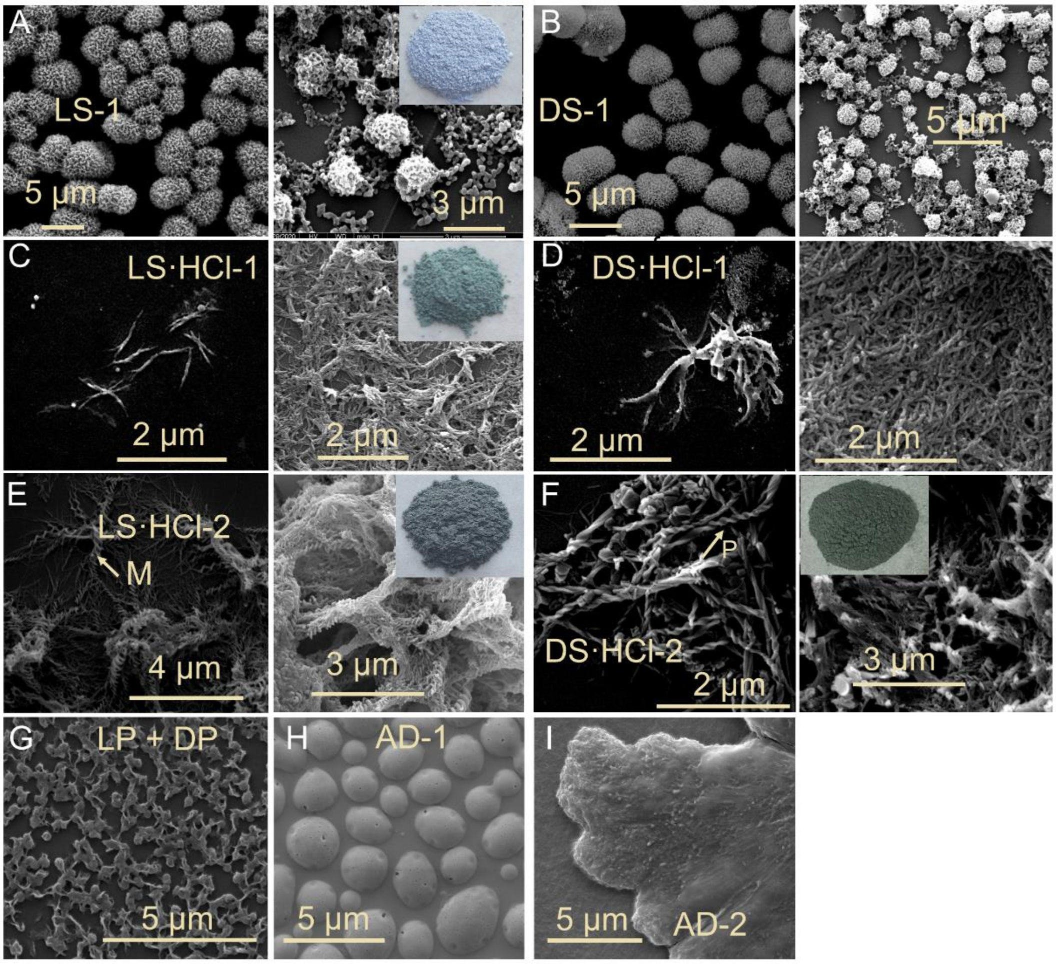

Figure S14. L/DP assemblies with different morphologies. Related to Figure 1. Scanning electron microscopy (SEM) images of (A) LP assemblies (LS-1) from HFIP, (B) DP assemblies (DS-1) from HFIP, (C) LP assemblies (LS·HCl-1) from HFIP-HCl, (D) DP assemblies (DS·HCl-1) from HFIP-HCl, (E) LP assemblies (LS·HCl-2) from 1-pentanol-HCl, (F) DP assemblies (DS·HCl-2) from 1-pentanol-HCl. (A-F), Left: Dropping the solution containing LP or DP

assemblies to silicon wafer; Right: Coating silicon wafers by dip-coating with solution containing LP or DP assemblies. Insets show LP or DP assemblies with different colors: LS-1 (light purple), LS·HCl-1 (grayish green), LS·HCl-2 (grayish black) and DS·HCl-2 (grayish black). SEM images of (G) mixed LP and DP (mole ratio of 1:1) from 1-pentanol-HCl. Dropping the solution containing the assemblies to the silicon wafer. SEM images of AD assemblies from HFIP (H) and 1-pentanol (I).

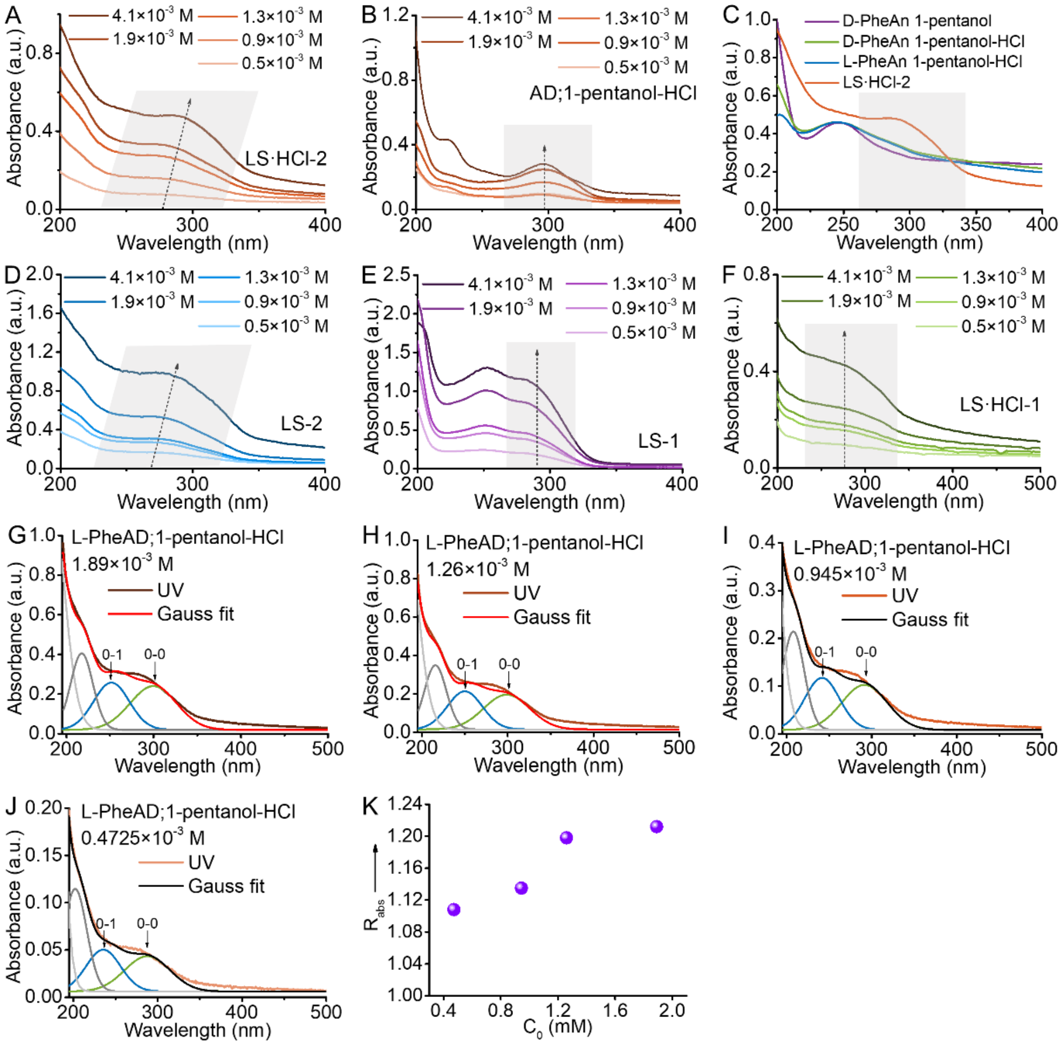

Figure S15. Ultraviolet-visible (UV-Vis) spectra of L/DP assemblies and AD molecules. Related to Figure 2. UV-Vis spectrum of (A) LP with increasing concentration in 1-pentanol-HCl mixture, (B) AD molecules with increasing concentration in 1-pentanol-HCl mixture, (C) L or D-PheAn (chemical structures in Figure. S16E) assemblies and LP assemblies (3 mg/mL). LP in 1-pentanol-HCl mixture shows an ultraviolet absorption peak at about 293 nm. No ultraviolet absorption peak (around 293 nm) is found when L/D-PheAn are dispersed in 1-pentanol-HCl (or 1-pentanol). The absorption band around 293 nm should be associated with AD segments due to their conjugated properties. (D-F), UV-Vis spectrum of LP with increasing concentration in 1-pentanol (D), in HFIP (E), and in HFIP-HCl (F). (G-J), Gaussian deconvolution of the UV-vis spectrum of LS·HCl in Figure S15 A. The confidence interval R^2 of the fitted curve are 0.9944, 0.9954, 0.9954, 0.9976, respectively. (K) A^{0-0} /A^{0-1} ratio (R_{abs}) for LS·HCl in Figure S15 G-J at RT.

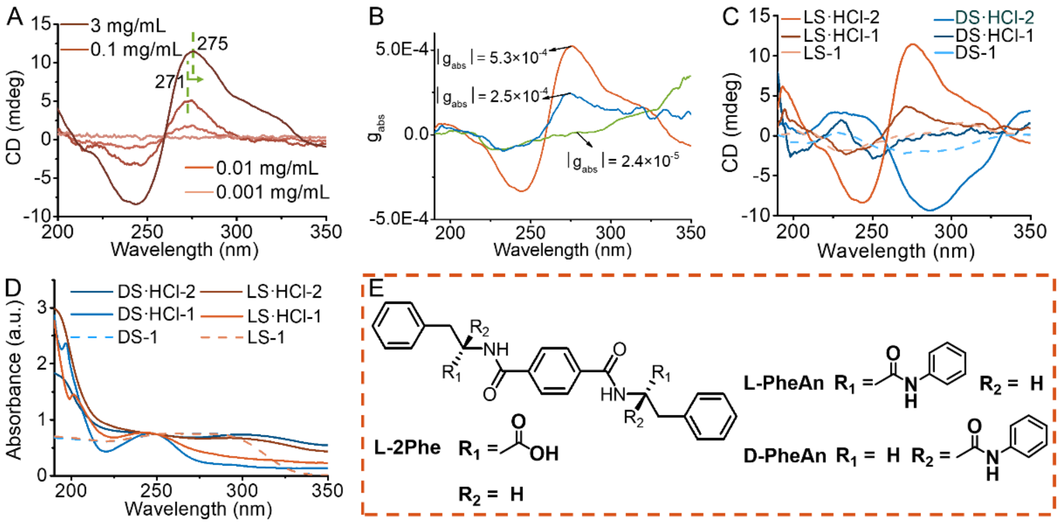

Figure S16. Circular dichroism (CD) spectra of L/DP assemblies and AD molecules. Related to Figure 2. CD spectra of (A) LS·HCl-2 with increasing concentration of LP. Corresponding g_{abs} spectra of (B) LS·HCl-2, LS·HCl-1 and LS-1. CD spectra of (C) LP or DP assemblies are recorded in UV region (190-350 nm) by a 0.1 mm quartz cuvette at a concentration of 3 mg/mL. UV spectrum (corresponding to Figure. S16C CD spectra) of (D) LP or DP assemblies. Chemical structures of (E) L-PheAn, D-PheAn, and L-2Phe.

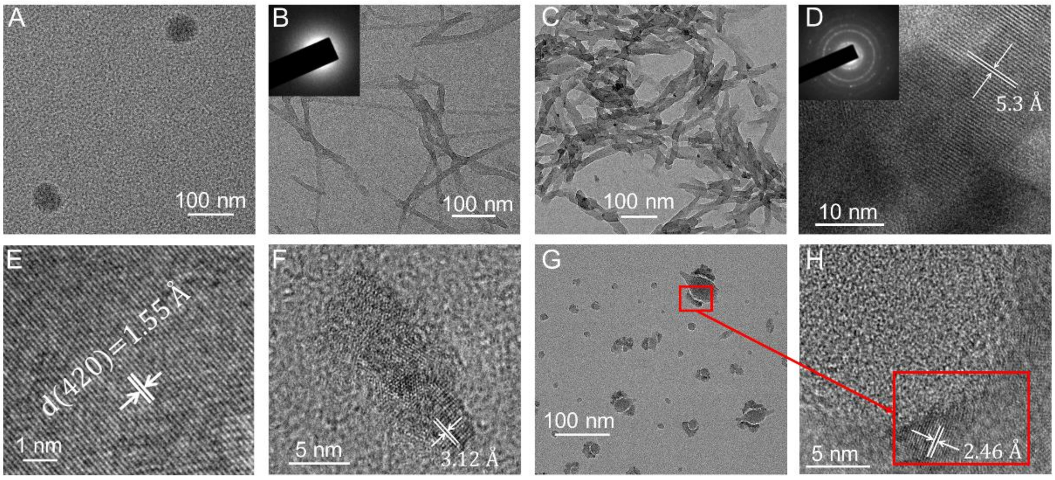

Figure S17. TEM of LP assemblies. Related to Figure 2. TEM images of (A-D) LP assemblies (LP assemblies dispersed in solvent and let stand for 24 hours). Inset shows the selected area electron diffraction patterns. (A) LS-1; (B) LS·HCl-1; (C) LS·HCl-2; Bright-field TEM images of (D) LS·HCl-2. Bright-field TEM images of (E, F) LS·HCl-2 (LP in 1-pentanol-HCl and stand for 1 week), (G, H) LS·HCl-2 (LP in 1-pentanol-HCl and let stand for 1 hour).

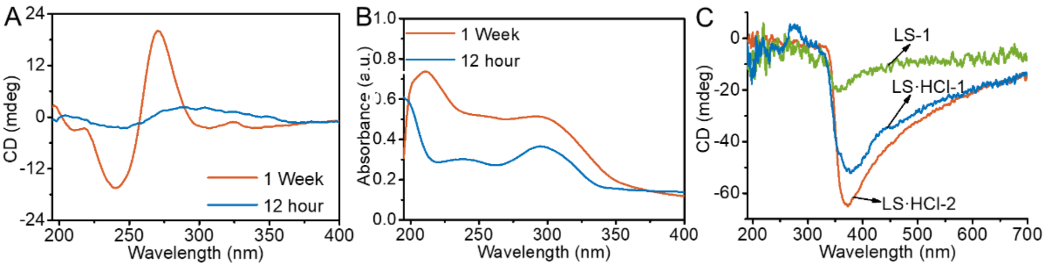

Figure S18. CD spectra of LP assemblies. Related to Figure 2. CD spectra (A) and corresponding UV absorption spectra (B) of LS·HCl-2. (LP dispersed in 1-pentanol-HCl and let stand for different times). CD spectra of (C) LS-1, LS·HCl-1 and LS·HCl-2 in solid state.

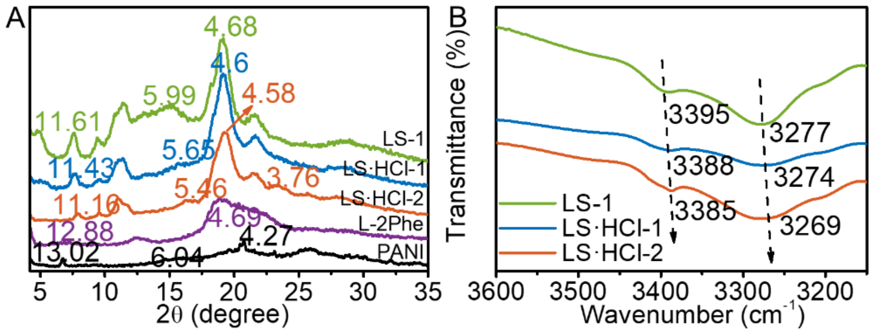

Figure S19. Powder X-ray diffraction (XRD) patterns and Fourier-transform infrared spectroscopy (FT-IR) of LP assemblies. Related to Figure 2. XRD patterns of (A) LS-1, LS·HCl-1, LS·HCl-2, L-2Phe and PANI. FT-IR of (B) LS-1, LS·HCl-1, LS·HCl-2.

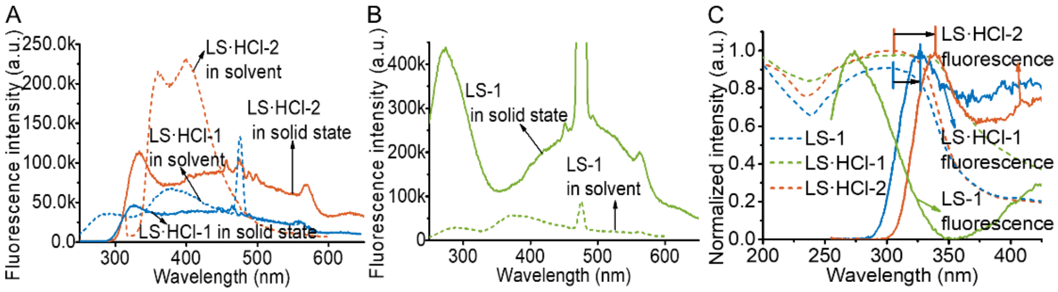

Figure S20. Optical properties of LP assemblies. Related to Figure 2. Fluorescence emission spectra of (A) LS·HCl-1, LS·HCl-2 and (B) LS-1 in the dispersing solvent (3mg/mL; 3mL) and in dried form (50 mg tablet sample). Fluorescence emission spectra (solid line) and UV absorption spectrum (dotted line) of (C) LP assemblies in solid state.

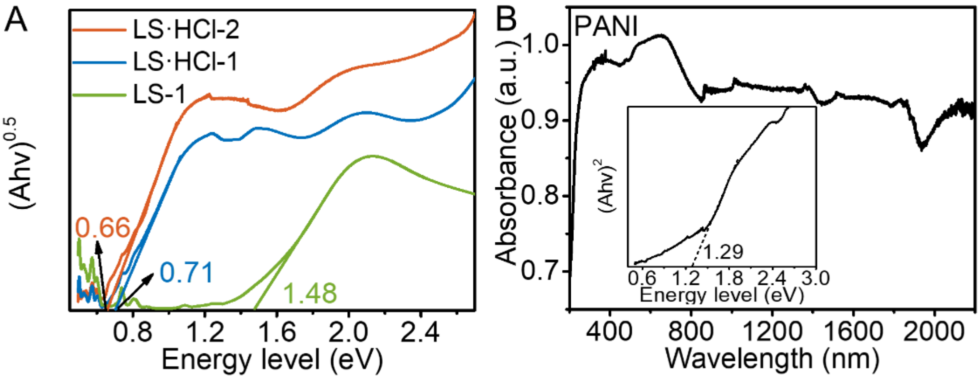

Figure S21. Optical gap. Related to Figure 2. Optical gap of (A) LP assemblies. Diffuse reflectance ultraviolet-visible-near-infrared (UV-Vis-NIR) spectroscopy of (B) PANI. (Inset shows optical gap of PANI).

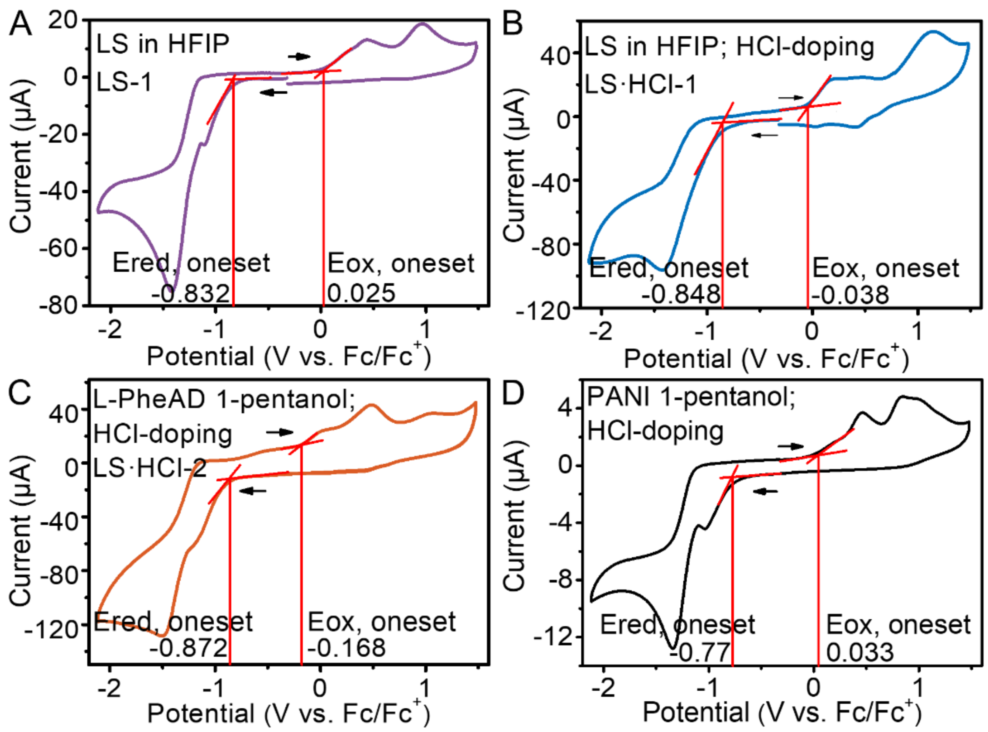

Figure S22. Electrochemical gaps of LP assemblies and PANI. Related to Figure 2. (A-D) Cyclic voltammograms (CV) were recorded on LP assemblies or PANI drop-casted onto glassy carbon electrode (GCE) with 0.1 M tetrabutylammonium hexafluorophosphate (Bu_4NPF_6) as supporting electrolyte in dichloromethane. (A) LS-1; (B) LS·HCl-1; (C) LS·HCl-2; (D) PANI. LP assemblies modified GCE or PANI modified GCE electrode were used as the working electrode. Ag/AgCl electrode as the reference electrode. Eox,onset and Ered,onset determined from the onset potentials of the oxidation and reduction waves, respectively. Scan speed = 50 mV/s. All CV were calibrated against Fc/Fc^+ redox couple in respective solvents.

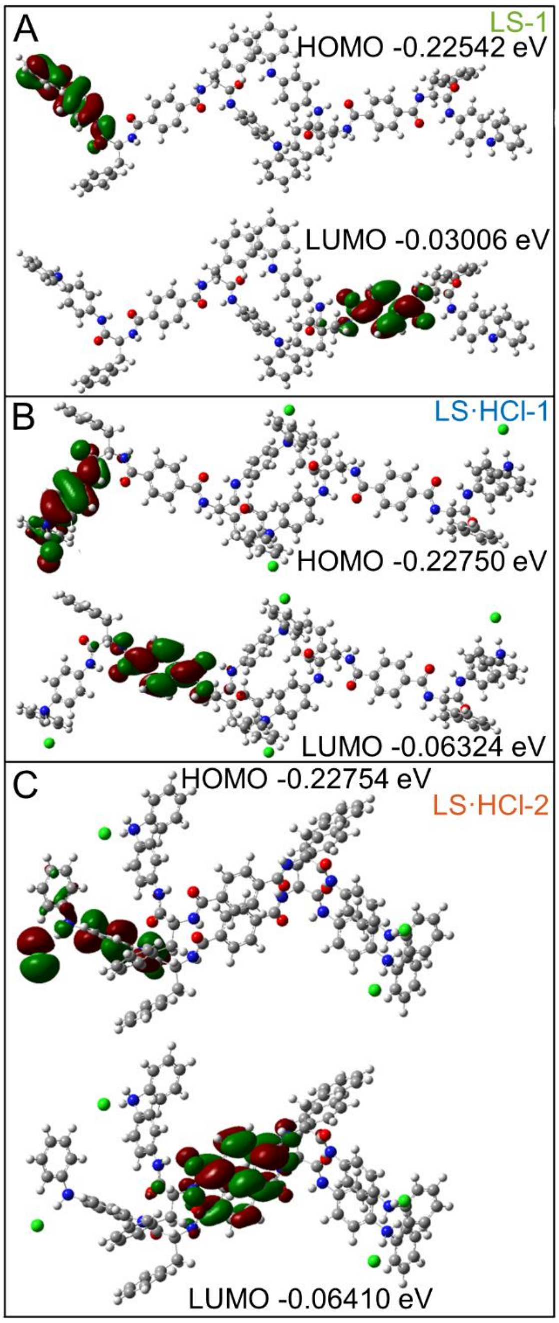

Figure S23. The energies of HOMO, LUMO levels of LP assemblies. Related to Figure 2. The energies of HOMO, LUMO levels, and the lowest energy state of LP dimer arrangement in (A) LS-1, (B) LS·HCl-1 and (C) LS·HCl-2.

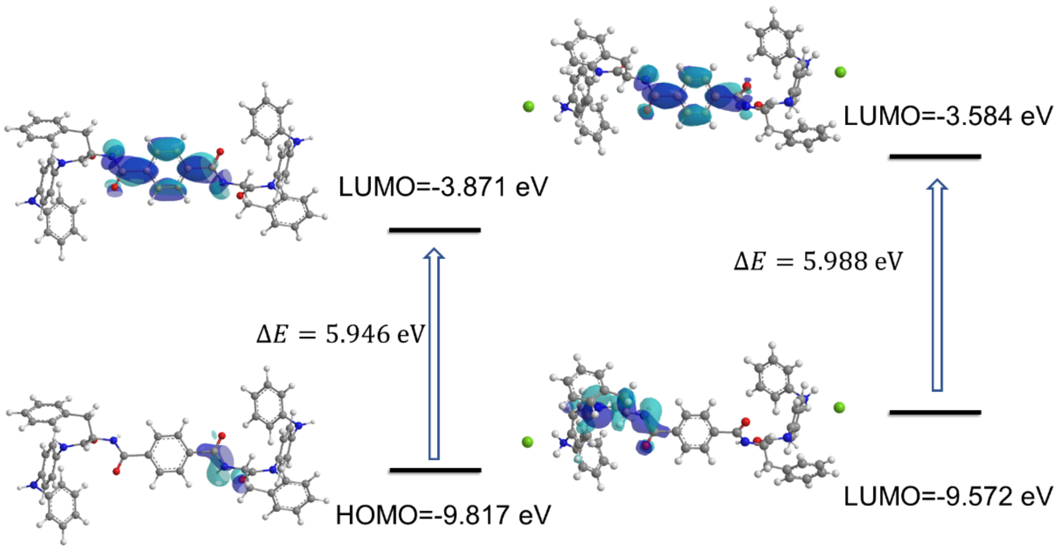

Figure S24. The energies of HOMO, LUMO levels of LP single molecule. Related to Figure 5. The energies of HOMO, LUMO levels of LP without HCl doping (left) and with HCl doping (right). An isovalue of 0.02 was used for drawing the orbital surfaces.

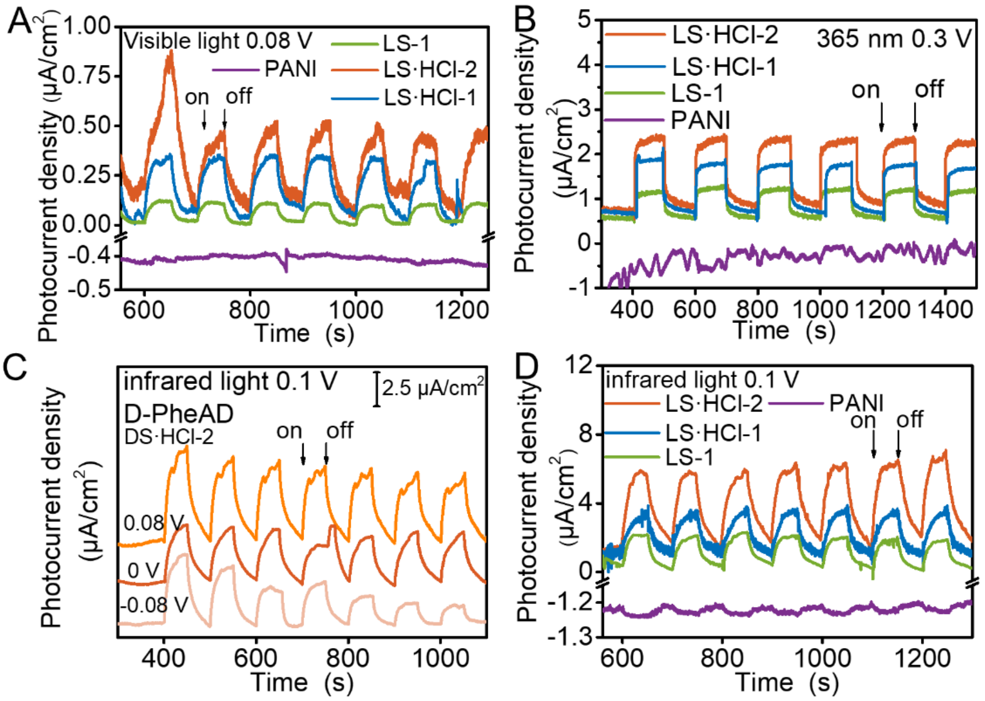

Figure S25. The time-dependent photoresponse of LP assemblies and PANI. Related to Figure 3. Photoresponse of PANI and LP assemblies under (A) 400-780 nm illumination, under (B) 365 nm illumination and under (D) 800-1000 nm illumination. Photoresponse of (C) DS·HCl-2 under 800-1000 nm illumination. (A-D), Electrical properties of L/DP assemblies can also be seen from the experimental results of time-dependent photoresponse. LS-1, LS·HCl-1, LS·HCl-2, and DS·HCl-2 exhibit photoconductivity across a wide spectral range from ultraviolet light to near-IR and magnitude of current could be switched between on and off with the light. It is observed that the enhanced photocurrent density is obtained for LS·HCl-2, which can probably ascribe to the reduced bandgap[1,2].

1.3. Effect of HCl doping on the energy band structure of LP assemblies

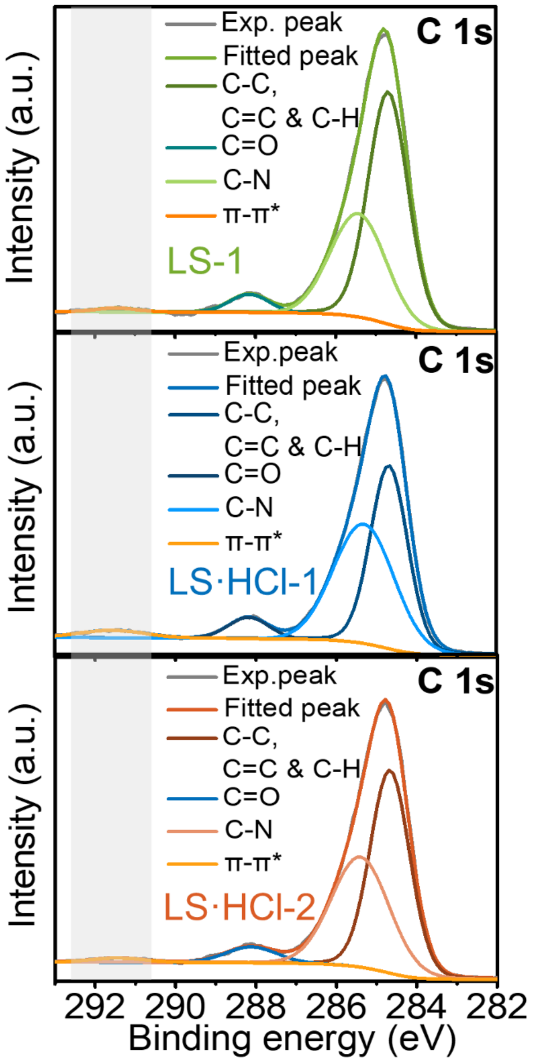

Figure S26. The high-resolution X-ray photoelectron spectroscopy (XPS) spectra of LP assemblies. Related to Figure 3. XPS spectra of C1s for LS-1, LS·HCl-1, LS·HCl-2.

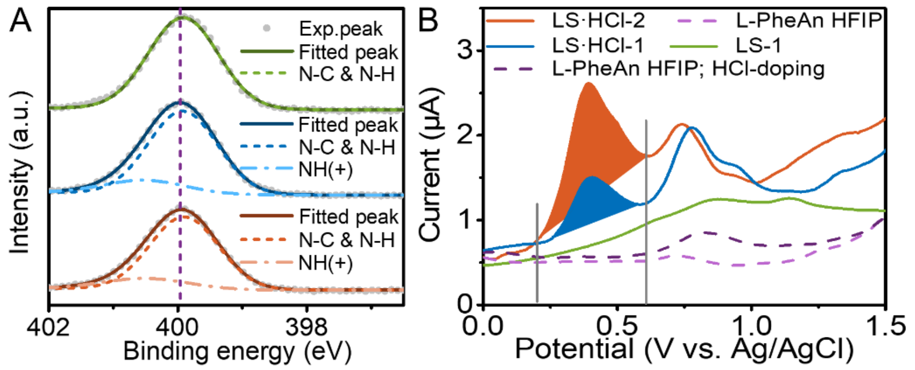

Figure S27. Effect of HCl doping on the XPS spectra and CV curves of LP assemblies. Related to Figure 3. N 1s XPS spectrum collected from (A) LP assemblies. By high-resolution XPS, there is a ~0.1 eV shift of N 1s spectra to higher energy region for LS·HCl-1 (bule line) or LS·HCl-2 (red line) than that of LS-1 (green line). The N 1s spectra of LS·HCl-1 or LS·HCl-2 can be deconvoluted into two distinct peaks centered at ~399.9 and ~400.5 eV, designating to the neutral and protonated amino groups[3], respectively. Linear potential sweep voltammetry (LSV) curves for (B) LP assemblies and L-PheAn (chemical structures in Figure S16E) assemblies. Three-electrode system: a platinum foil (1 × 5 mm) was used as the auxiliary electrode and an Ag/AgCl electrode as the reference electrode; scan rate 50 mV/s; electrolyte:

0.1 M Bu_4NPF_6 as the supporting electrolyte in dichloromethane. By LSV experiment, there are no new redox peak and clear shifts of the redox potentials of LS-1 and L-PheAn compared with LS·HCl-2 or LS·HCl-1, implying that the site of acidification of LP should locate in -NHPh group of AD segment.

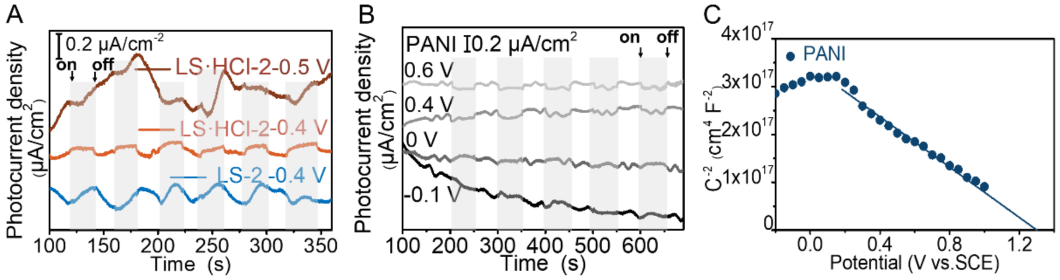

Figure S28. Photoresponsive current of LS·HCl-2, LS-2 and PANI at different voltage bias. Related to Figure 3. Photoresponsive current of (A) LP assemblies (LS·HCl-2, LS-2) and (B) PANI at different voltage bias under the illumination of 365 nm. Mott-Schottky measurements of (C) PANI in 0.2 M KCl at an applied frequency of 5 kHz. M-S approach manifest that PANI is p-type semiconductor in the presence of HCl.

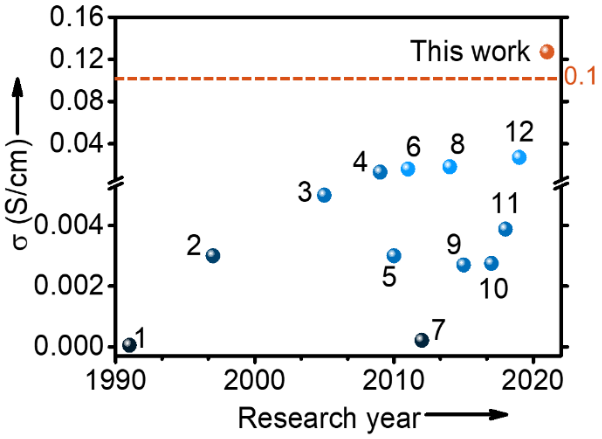

Figure S29. A summary of the conductivity of aniline tetramer (AT), AT derivatives from references, and LS·HCl-2 (this work). Related to Figure 3. 1: AT powder pressed pellet (ref.[4]); 2: AT powder compressed pellet (ref.[5]); 3: complexes of surfactant and AT (ref.[6]); 4: AT powder compressed pellet (ref.[7]); 5: AT powder compressed pellet (ref.[8]); 6: AT films (ref.[9]); 7: Single-crystalline microplate structures of AT. (ref.[10]); 8: Electroactive microcapsules of AT (ref.[11]); 9: AT-PTAB film (ref.[12]); 10: F_{127}/AT-PEI composites (ref.[13]); 11: AT - PTSA (ref.[14]); 12: Dex-AT/CECS hydrogel (ref.[15]).

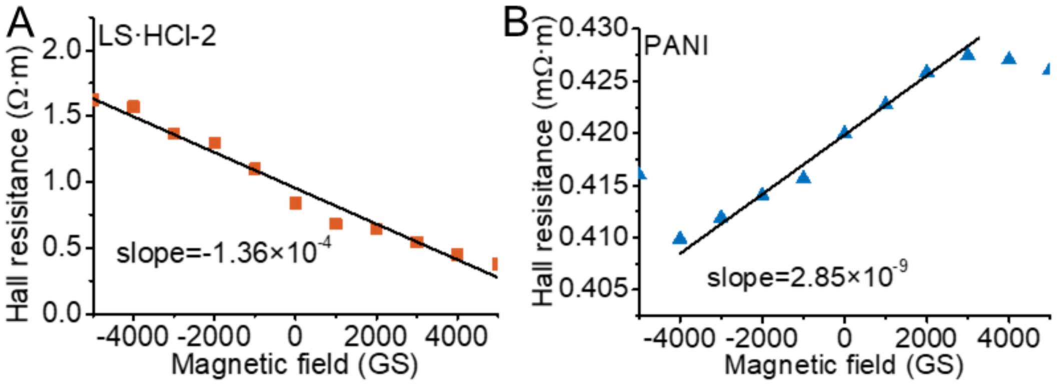

Figure S30. Hall resistance of LS·HCl and PANI. Related to Figure 4. Hall resistance dependence on the magnetic field at 298 K for (A) LS·HCl and (B) PANI.

$$R_H(LS \cdot HCl - 2) = \frac{V_H \times d}{Is \times B} = -10^8 \times 1.36 \times 10^{-4} \times 0.1 = -1.36 \times 10^3\ (cm^3 \cdot C^{-1})$$

$$R_H(PANI) = \frac{V_H \times d}{Is \times B} = 10^8 \times 2.85 \times 10^{-9} \times 0.15 = 0.042\ (cm^3 \cdot C^{-1})$$

$$\mu_H(LS \cdot HCl - 2) = \frac{|R_H|}{\rho} = 172.72\ cm^2 \cdot V^{-1} \cdot S^{-1}$$

$$\mu_H(PANI) = \frac{|R_H|}{\rho} = 0.0053\ cm^2 \cdot V^{-1} \cdot S^{-1}$$

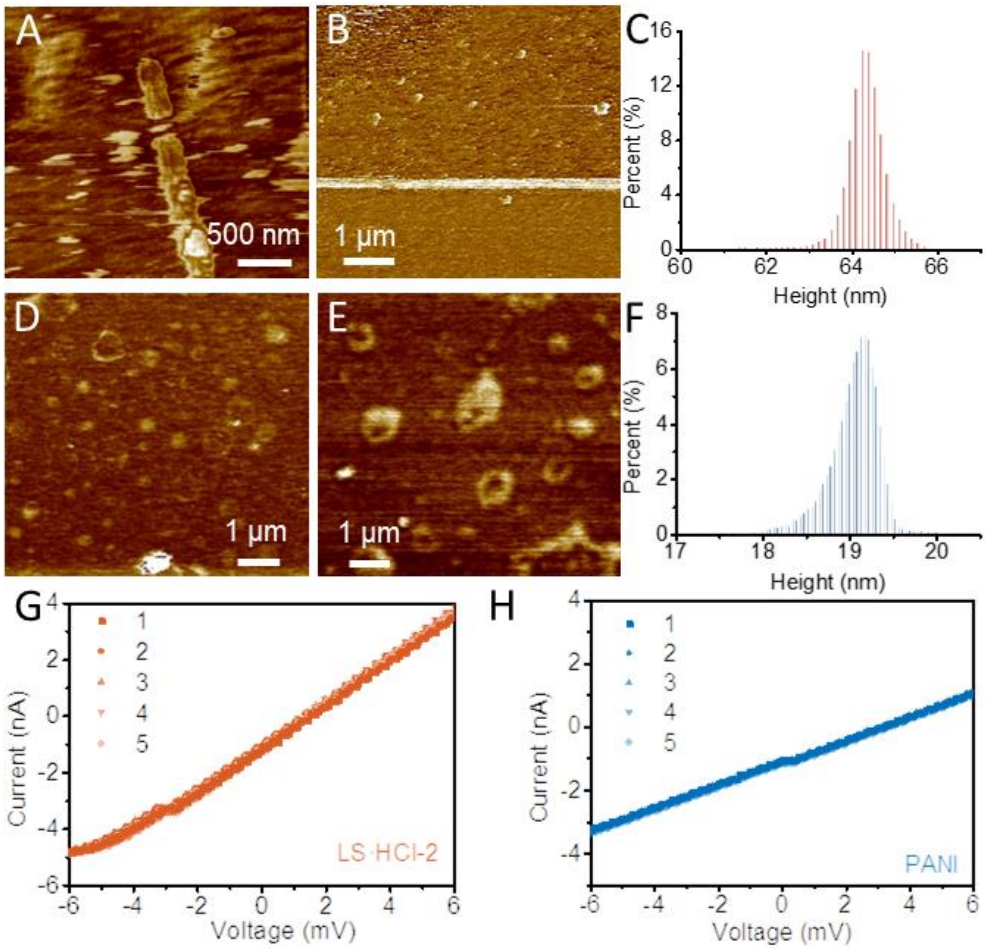

Figure S31. Conductivity estimation of the LP nanofiber and PANI nanoparticle from CAFM analysis. Related to Figure 4.

Typical AFM topographic image (A, B) and corresponding height histogram (C) of LS·HCl-2 nanofibers on Si wafer within the scanned area. Typical AFM topographic image (D, E) and corresponding height histogram (F) of PANI nanoparticles on Si wafer within the scanned area. Typical *I-V* curves obtained for (G) LS·HCl-2 nanofibers and PANI nanoparticles. Schematic of the conductive-AFM measurement setup for (H) LS·HCl-2 nanofibers probing current.

The conductivity values of LP nanofiber and PANI nanoparticle were calculated from the *I-V* curves obtained from conductive AFM measurements using the equations below:

R = V / I; ρ = R · (A/l); σ = 1/ρ

where R is resistance, V is voltage, I is current, ρ is resistivity, l is channel length, A is channel cross-sectional area, and σ is conductivity. The conductivity values shown below are the average of 10 measurements for each sample.

Since the length of LS·HCl-2 nanofibers varies considerably and the PANI particles have an irregular morphology, choosing the thickness of nanoparticles as the channel length which is also the shortest conduction path, the pathway shown in Figure S29H.

LS·HCl-2 nanofibers:

AFM tip area (A_{AFM}) = 314 nm^2 (AFM tip diameter= 20 nm)

LS·HCl-2 nanofiber average height (l_{avg}) = 64.3 nm

Average resistance from 10 mesaurements: R_{avg} = 1.278×10^6 Ω

$\rho_1 = R_{avg} \cdot (A_{AFM}/l_{avg}) = (1.278\times10^6\ \Omega) \cdot (314\ nm^2/\ 64.3\ nm) \cdot (10^{-7}\ cm/nm) = 0.624\ \Omega \cdot cm$
$\sigma_1 = 1/\rho_1 = 1.603$ S/cm

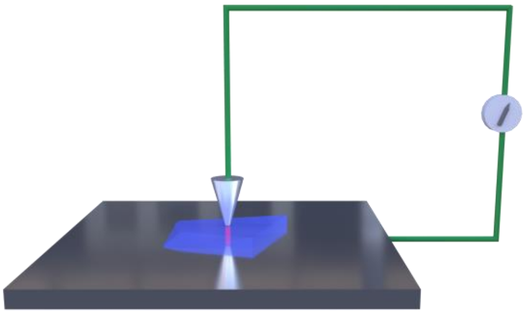

Figure S32. Conductivity estimation of the PANI nanoparticles from CAFM analysis. Related to Figure 4. Schematic of the conductive-AFM measurement setup for PANI nanoparticles probing current. The red line represents the assuming preferred carrier transport pathway of carriers from the cAFM tip to the bottom Si wafer.
PANI nanoparticles:
AFM tip area (A_{AFM}) = 314 nm^2 (AFM tip diameter= 20 nm)
PANI average height (l_{avg}) = 19.2 nm
Average resistance from 10 mesaurements: $R_{avg} = 1.69\times10^6\ \Omega$
Assuming preferred carrier transport pathway as shown in Fig. S31:
$\rho_1 = R_{avg} \cdot (A_{AFM}/l_{avg}) = (1.69\times10^6\ \Omega) \cdot (314\ nm^2/\ 19.2\ nm) \cdot (10^{-7}\ cm/nm) = 2.764\ \Omega \cdot cm$
$\sigma_1 = 1/\rho_1 = 0.362$ S/cm

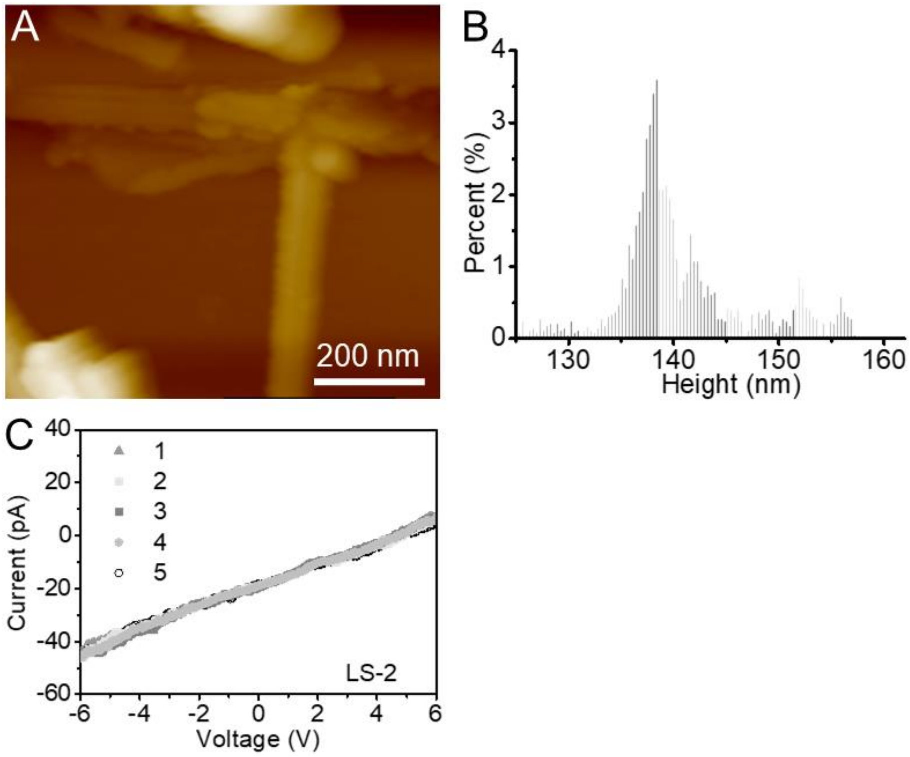

Figure S33. Conductivity estimation of the LS-2 nanofibers from CAFM analysis. Related to Figure 4.
Typical AFM topographic image (A) and corresponding height histogram (B) of LS-2 nanoparticles on Si wafer within the scanned area. Typical *I-V* curves obtained for (C) LS-2 nanofibers.
AFM tip area (A_{AFM}) = 314 nm^2 (AFM tip diameter= 20 nm)
LS-2 average height (l_{avg}) = 138 nm

Average resistance from 10 mesaurements: $R_{avg} = 2.13\times10^{11}\ \Omega$
Assuming preferred carrier transport pathway as shown in Fig. S31:
$\rho_1 = R_{avg}\cdot(A_{AFM}/l_{avg}) = (2.13\times10^{11}\ \Omega)\cdot(314\ nm^2/\ 138\ nm)\cdot(10^{-7}\ cm/nm) = 4.85\times10^3\ \Omega\cdot cm$
$\sigma_1 = 1/\rho_1 = 2.06\times10^{-4}$ S/cm

1.4. Electrochemical study of LP assemblies and PANI as well as stability analysis

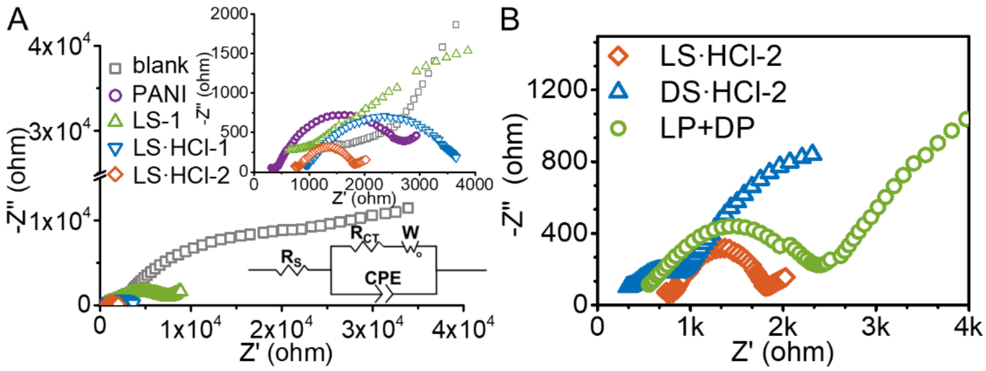

Figure S34. Electrochemical alternate impedance spectroscopy (EIS) of LP assemblies and PANI. Related to Figure 5. EIS of (A) LP assemblies and PANI. LP assemblies or PANI/glass (the size of glass electrode is 6 × 10 mm) electrode was used as the working electrode (the copper wire as a conductor). Inset: equivalent circuit of LP assemblies and PANI coated glass electrodes. Nyquist plots of impedance spectra for (B) LP assemblies, DP assemblies, and LP+DP (mole ratio of 1:1).

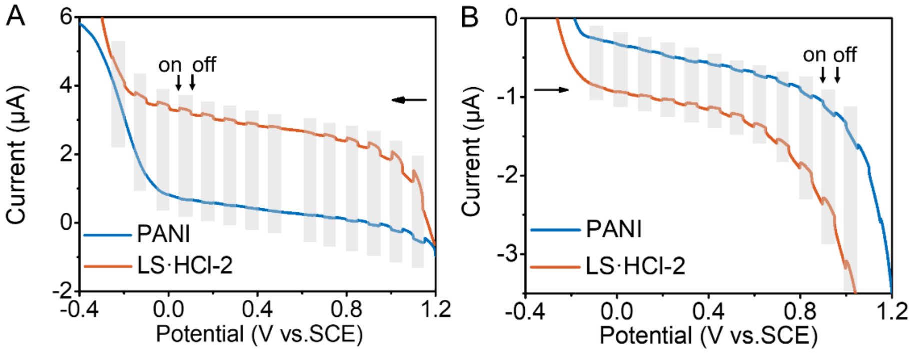

Figure S35. Linear potential sweep voltammetry (LSV) curves of LS·HCl-2 and PANI. Related to Figure 5. linear potential sweep voltammetry (LSV) curves of (A) LS·HCl-2 and PANI films on glass substrate from 1.2 to -0.3 V (vs SCE). LSV curves of (B) LS·HCl-2 and PANI films on glass substrate from -0.3 to 1.2 V (vs SCE).

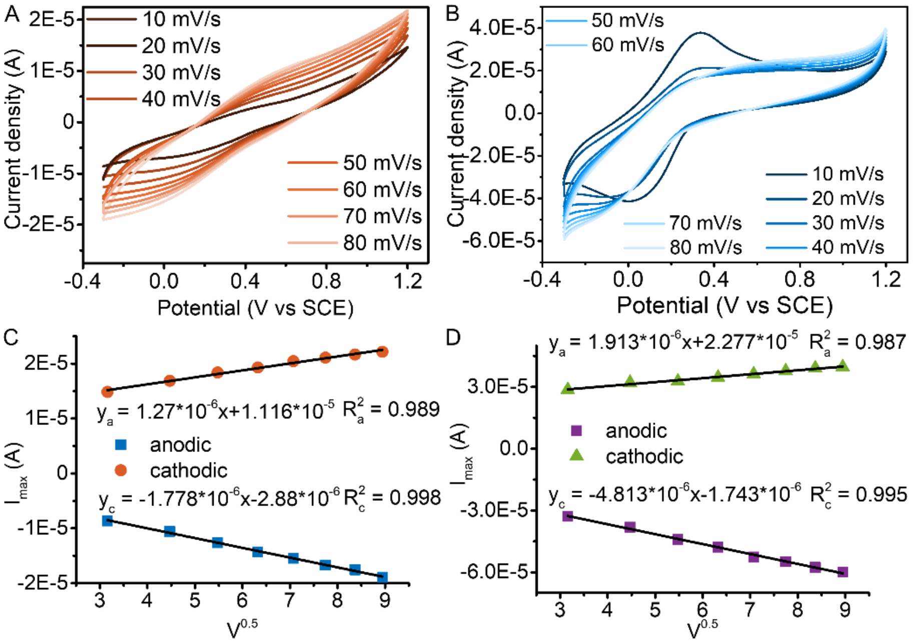

Figure S36. CV curves of LS·HCl-2 and PANI. Related to Figure 5. CV of (A) LS·HCl-2 and (B) PANI electrodes under varying scan rates. Plot of maximum current peak vs square root of scan rate of (C) LS·HCl-2 and of (D) PANI

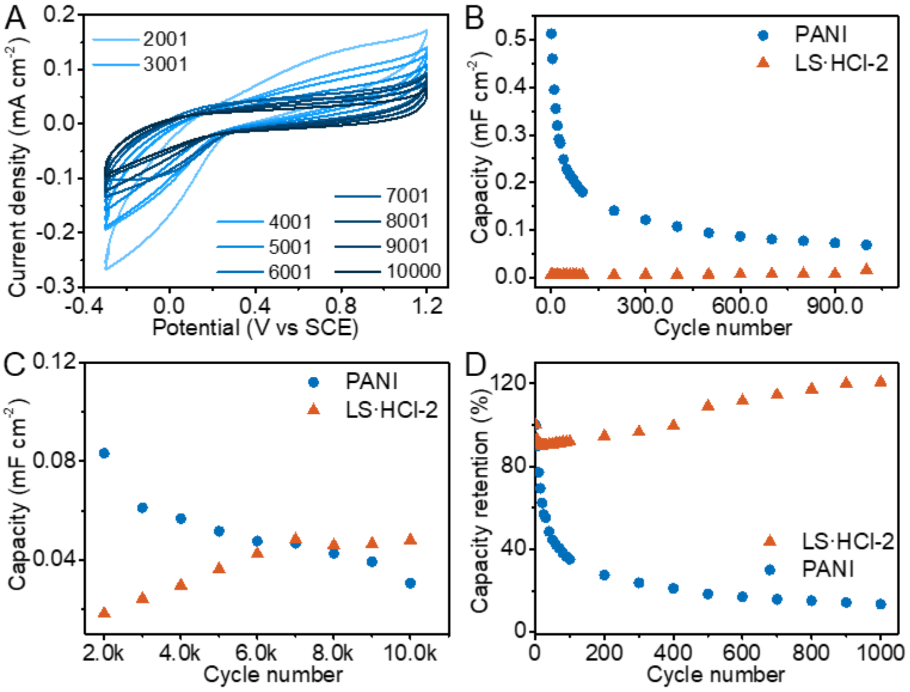

Figure S37. CV curves and the corresponding areal capacitance of PANI. Related to Figure 5. CV of (A) PANI at the scan rate of 500 mV/s. A plot of (B and C) areal capacitance, and (D) capacitance retention vs. cycle numbers.

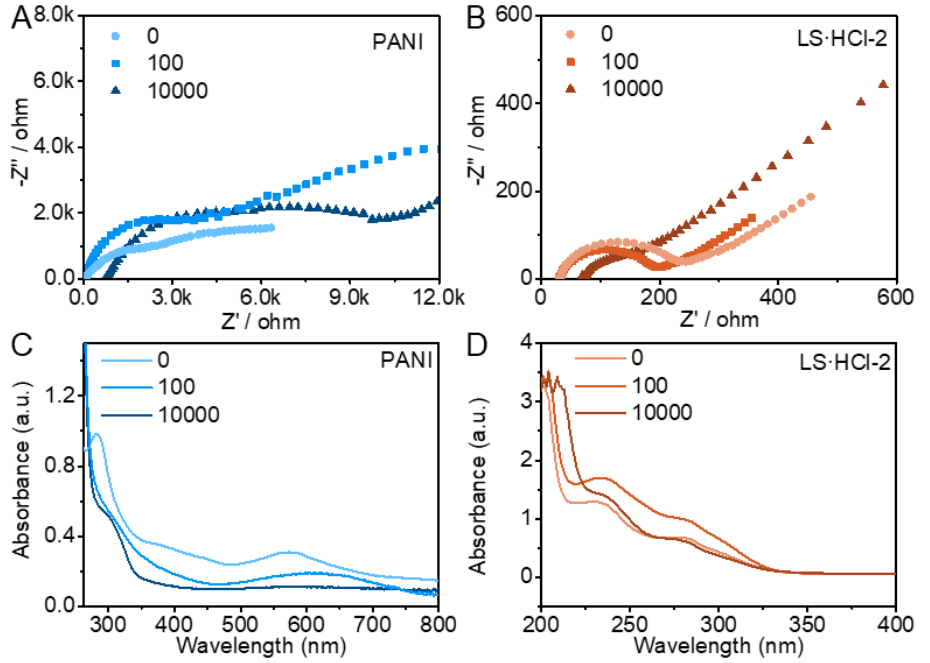

Figure S38. EIS of PANI and LS·HCl-2 after CV scanning. Related to Figure 5. Electrochemical alternate impedance spectroscopy (EIS) of (A) PANI and (B) LS·HCl-2 after 100 cycles and 10000 cycles. UV-Vis spectra of (C) PANI and (D) LS·HCl-2 after 100 cycles and 10000 cycles.

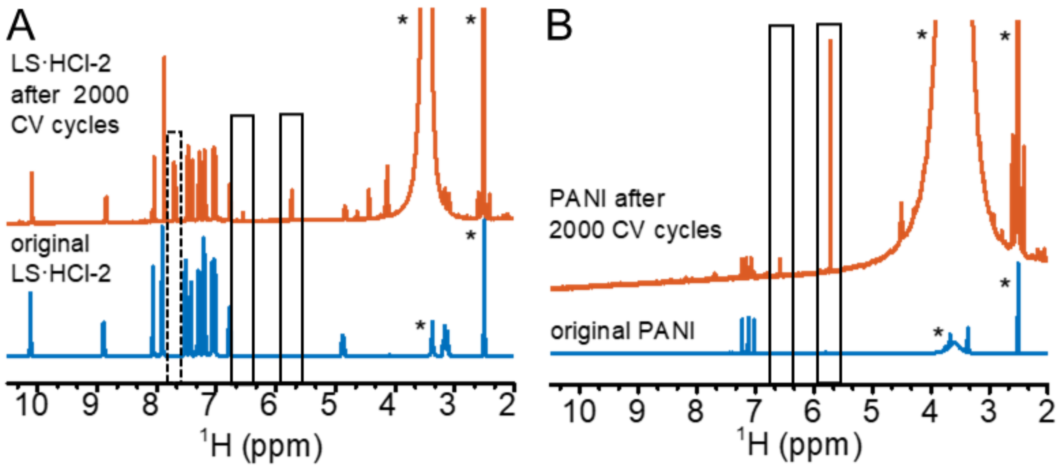

Figure S39. ^{1}H NMR spectrum of LS·HCl-2 and PANI before and after CV scanning. Related to Figure 5. ^{1}H NMR spectrum of (A) LS·HCl-2 and (B) PANI before and after 2000 cycles of CV scanning.

The ^{1}H NMR spectra of LS·HCl-2 shows new peak appear at 7.63-7.74 ppm ascribes to the quinone ring in oxidized AD unit. The new peaks with ^{1}H-chemical shifts around 6.53 and 5.72 ppm appear in the spectra of both LP and PANI after 2000 cycles. These peaks should belong to the main products of LS·HCl-2 and PANI degradation, soluble species such as benzoquinone (BQ) and hydroquinone (HQ), and/or insoluble fragments containing quinonic functional groups at their ends [16]. It is found that intensity of the peaks of PANI at 6.53 and 5.72 ppm are much stronger than that of LS·HCl-2, indicating that PANI contained more degradation products.

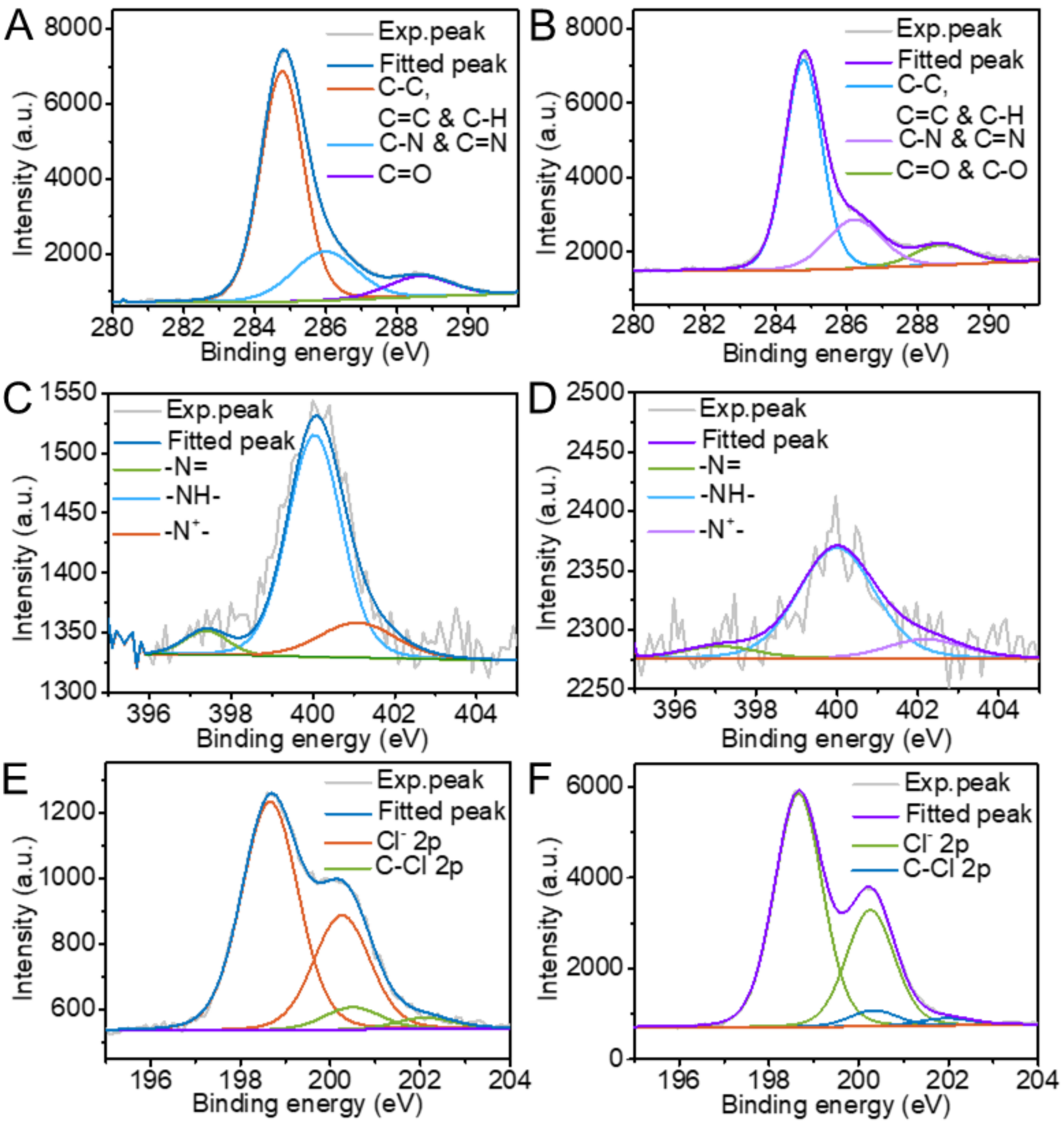

Figure S40. High-resolution XPS spectra of LS·HCl-2 and PANI after 2000 cycles of CV scanning. Related to Figure 5. C 1s XPS spectra of (A) LS·HCl-2 and (B) PANI. N 1s XPS spectra of (C) LS·HCl-2 and (D) PANI. Cl 2p XPS spectra of (E) LS·HCl-2 and (F) PANI.

The C 1s peaks of LS·HCl-2 and PANI can be deconvoluted into three components: ~284.8 eV related to C-C, C=C and C-H; ~285.9 eV related to C-N and C=N; ~288.6 eV related to C-O and C=O. It is noteworthy that the proportion of C-O&C=O in PANI is higher than that in LP (Table S3), because the electrochemical degradation of PANI is more likely to occur than LS·HCl-2. The N 1s peaks of LS·HCl-2 and PANI can be deconvoluted into three components. There is a ~1 eV shift of N 1s peak at 402.3 eV to higher energy region for PANI than that of LS·HCl-2, designating to higher protonation effects in PANI than in LS·HCl-2, thus PANI is more susceptible to nucleophilic attack than LS·HCl-2. The Cl 2p spectra consist of two spin-orbit doublets: the first peaked at ~198.6 eV and ~200.3 eV which is attributed to the Cl^- ions; the second peaked at ~200.5 eV and ~202.1 eV which is attributed to the formation of Cl–C bond of PANI and LS·HCl-2.[17]

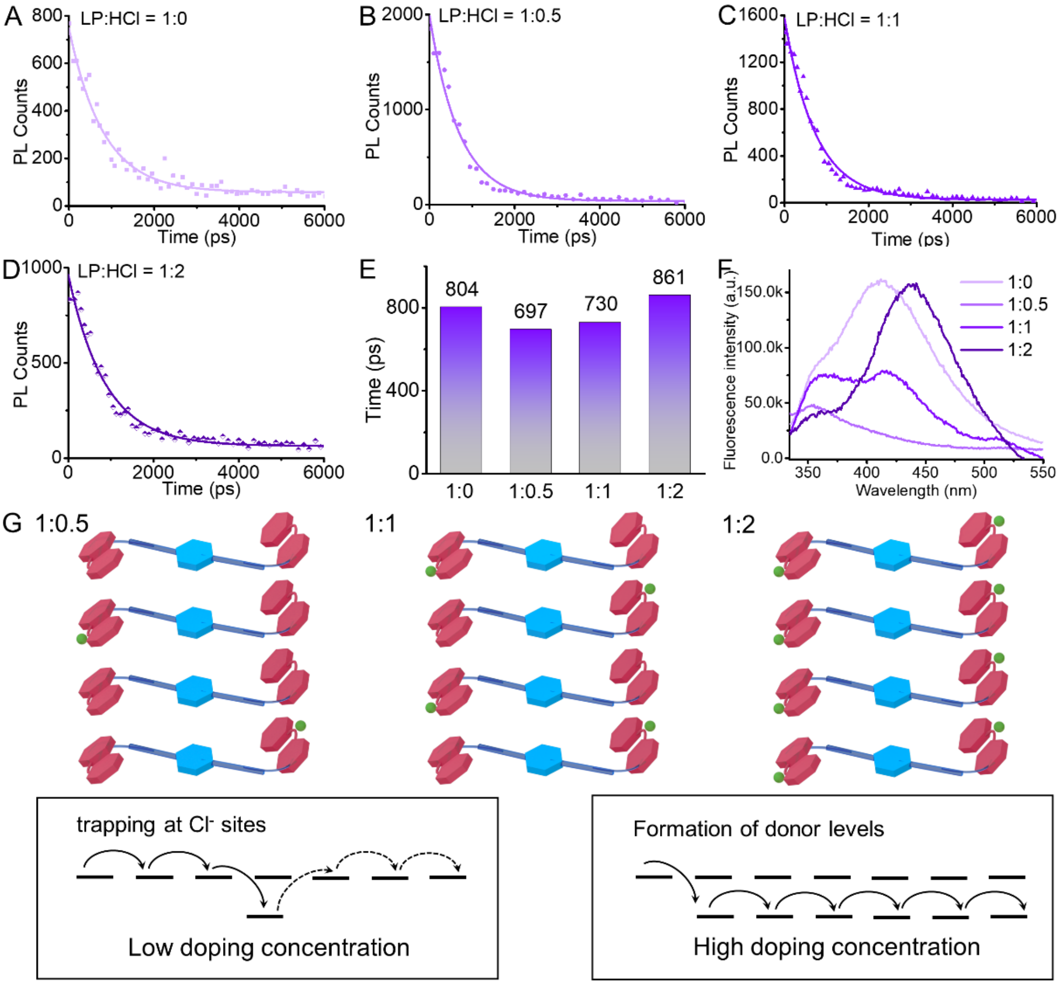

Figure S41. Time-resolved photoluminescence and Schematic diagram of charge transfer in the LP:HCl after light excitation. Related to Figure 5. Time-resolved photoluminescence and fitting analysis at a single wavelength: (A-D) LP:HCl molar ratio changes from 1:0 to 1:2. (E) The fluorescence lifetimes obtained in Figure S37A-S37D. (F) Fluorescence emission spectra of LP:HCl (1:X; X=0, 0.5, 1, 2). (G) Schematic diagram of charge transfer in the LP:HCl (1:X) after light excitation; Left: for low HCl doping concentration (X<2); Right: for high HCl doping concentration ($X \geqslant 2$), charge transfer via donor levels. Virtual arrow indicates there is a certain chance for the trapped carrier be thermally excited back to the conduction band.

As shown in Figure S41A-E, fluorescence lifetime declines to 697 ps as LP:HCl (1:X) molar ratio varies from 1:0 to 1:0.5, and increases from 697 to 861 ps as LP:HCl molar ratio varies from 1:0.5 to 1:2. When doping concentration is low (LP:HCl=1:X, X=0.5), there are small amount of AD units doped with HCL, photogenerated charge carriers undergo deep trapping at the Cl^- sites [18]. The decrease of LP:HCl (1:0.5) fluorescence lifetime and the decrease in fluorescence intensity of LP:HCl (1:0.5) steady-state photoluminescence spectrum (Figure S37F) correspond to this conclusion. When doping concentration is further increased (LP:HCl=1:X, X from 1 to 2), AD units are gradually doped with HCl and the donor energy level is gradually formed, then photogenerated charge carriers could transport through donor level, fluorescence lifetime increased. At the same time, steady-state photoluminescence spectrum shows that with the increase of the HCl doping concentration, the fluorescence intensity of LP:HCl (1:1) increased and the long-wavelength peak at 416 nm formed. Then, the peak at 416 nm is red-shifted to 440 nm and fluorescence intensity increased with further HCl doping, and the peak of LP:HCl (1:2) around 361 nm gradually decreased. These findings suggest the formation of a new energy transfer process due to the donor level formed[19].

1.5. LP assemblies act as stable conductive layer and sensing layer

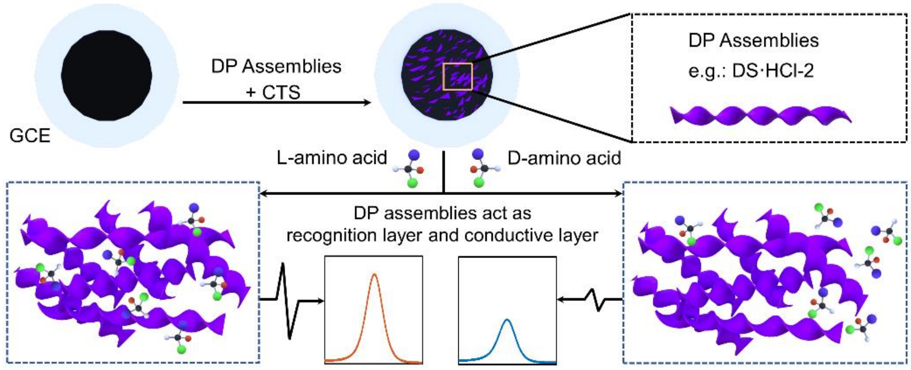

Figure S42. Schematic representation of the selective recognition platforms in electrochemical sensing. Related to Figure 6.

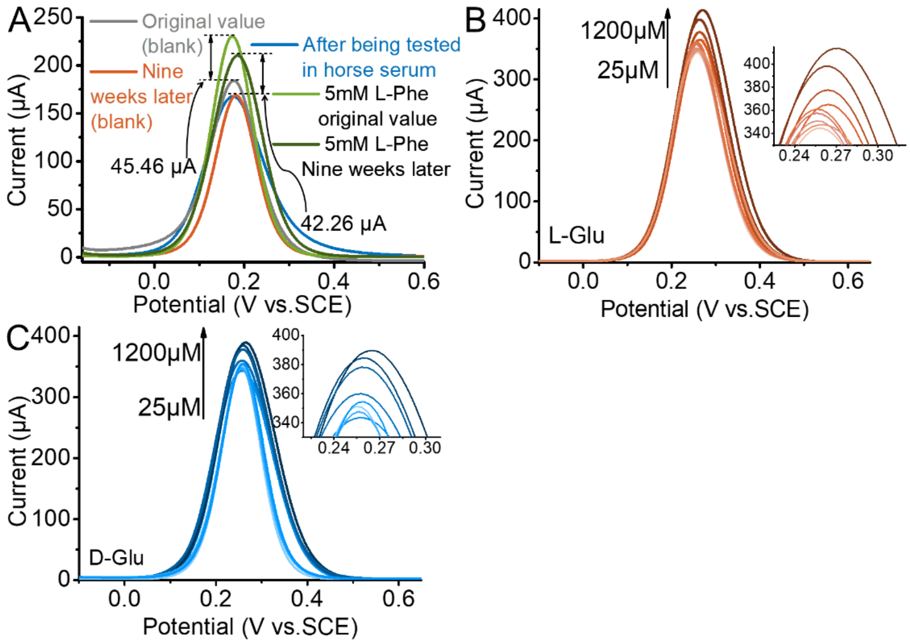

Figure S43. The response of DS·HCl-2 electrochemical sensors to glutamic acid (Glu) and the robustness of the sensors. Related to Figure 6. (A) DPVs of blank samples and 5 mM L-Phe by using fresh DS·HCl-2-CTS/GCE electrode (brown line and green line), aged DS·HCl-2-CTS/GCE electrode (stored at room temperature for 9 weeks; red line and dark green line), and the used DS·HCl-2-CTS/GCE electrode (after being tested in horse serum; green line) as working electrode, respectively. (electrolyte: 0.2 M KCl and 5mM $K_3[Fe(CN)_6]$ in water). DS·HCl-2 retains 93% of its initial response after a period of 9 weeks indicating the good reusability of the sensors. DPVs of L-Glu (B) and D-Glu (C) with different concentrations, respectively.

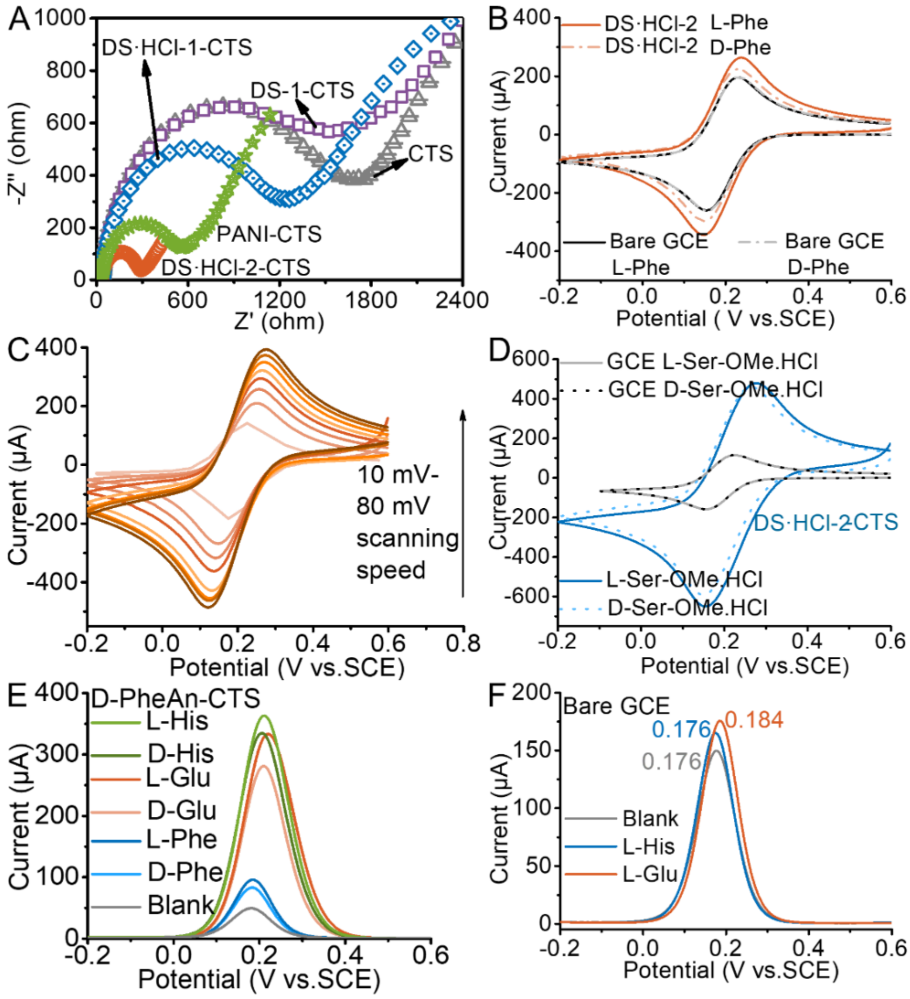

Figure S44. EIS and chiral recognition ability of DS assemblies and bare GCE. Related to Figure 6. (A) EIS of CTS (GCE modified with chitosan), DS-1-CTS (GCE modified by mixing DS-1 powders with CTS), DS·HCl-1-CTS (GCE modified by mixing DS·HCl-1 powders with CTS), DS·HCl-2-CTS (GCE modified by mixing DS·HCl-2 powders with CTS), PANI-CTS (GCE modified by mixing PANI powders from 1-pentanol-HCl with CTS). (B) CV of DS·HCl-2 electrochemical sensor (DS·HCl-2-CTS/GCE; red line) and GCE (black line). 5 mM L-phenylalanine (Phe) or D-Phe as test solvent. I_L of DS·HCl-2-CTS/GCE is larger than I_D, $I_L > I_D$. While bare GCE cannot recognize Phe isomers because of the overlapped oxidation and reduction peaks of L- and D-Phe, $I_D = I_L$. CV diagrams of (C) DS·HCl-2-CTS. DS·HCl-2-CTS can produce a reversible electrochemical process according to the increase of the peak potential difference (ΔEp) with the scan rate raise. CV of (D) DS·HCl-2 electrochemical sensor (DS·HCl-2-CTS/GCE; blue line) and GCE (gray line). 5 mM L-or D-Serine methyl ester hydrochloride (L/D-Ser-OMe.HCl) as test solvent. DPVs of (E) different amino acids at D-PheAn-CTS electrode (GCE modified by mixing D-PheAn powders from 1-pentanol-HCl with CTS). Current responses of L/D-glutamate (L/D-Glu; 5mM; red line), L/D-histidine (L/D-His; 5mM; green line), L/D-Phe (5mM; blue line), and blank (gray line). (F) Current responses of L-His (5mM; green line), L-Glu (5mM; red line), and blank (gray line) using GCE electrode. (Figure 5B) DPV anodic peak potential of Glu shifted in the positive direction than Phe. Compared with DPV anodic peak potential obtained from the blank control group, DPV anodic peak potential of His showed a negative shift. (E, F) On the contrary, neither D-PheAn modified electrode nor bare GCE can distinguish between positively and negatively charged amino acids. (B-F), Three-electrode system: a platinum foil (1 × 5 mm) was used as the auxiliary electrode and a saturated calomel electrode (SCE) electrode as the reference electrode; scan rate 50 mV/s; electrolyte: 0.2 M KCl and 5mM $K_3[Fe(CN)_6]$ in deionized water.

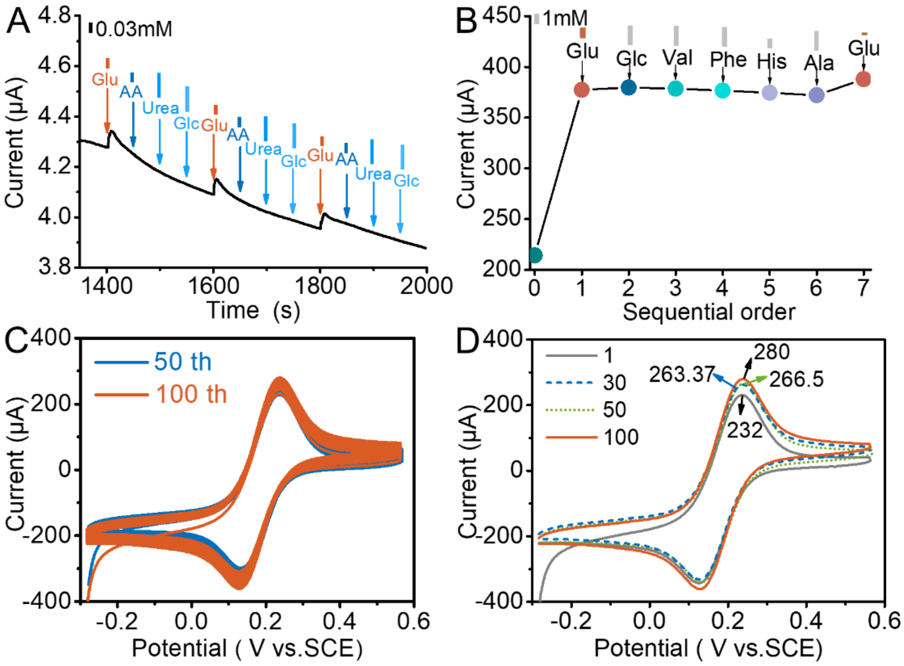

Figure S45. The selectivity and stability of DS·HCl-2-CTS/GCE. Related to Figure 6. The selectivity of (A) DS·HCl-2-CTS. The concentrations are 0.03 mM for L-Glu and ascorbic acid (AA), 0.06 mM for glucose (Glc) and urea. The interfering compounds are part of the major elements that affects the detection of L-Glu in blood. (Electrolyte: 0.2 M KCl and PBS buffer solutions at pH = 7.4). The specific recognition ability of DS·HCl-2 to Glu is attributable to the following reasons: As Glu is a dicarboxylic amino acid and it can be negative charged in deionized water. Two negatively charged carboxyl groups of Glu can bind with positive charge centers ($-NH_2^+-$) in the chain of AD segments and the electrical signal is achieved. DPV peak current of (B) different compounds at DS·HCl-2-CTS electrode. Glu (1 mM), Glc (2 mM), valine (Val; 2 mM), Phe (2 mM), His (1 mM), Ala (2 mM), Glu (0.25 mM) were added to solution in order. CV of (C) DS·HCl-2-CTS/GCE. The working electrode was cycled 50 and 100 times at a scan rate of 50 mV/s. DS·HCl-2 showed the variation of (D) peak current is less than 13.5%, 14.9%, 20.7% after 30, 50, 100 successive scans, respectively.

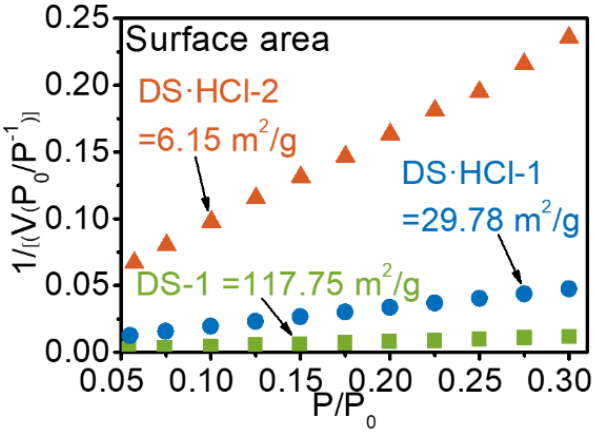

Figure S46. BET plot for DP assemblies. Related to Figure 6. DS-1 BET area: 117.75 m²/g; DS·HCl-1 BET area: 29.78 m²/g; DS·HCl-2 BET area: 6.15 m²/g. Meanwhile, N_2 adsorption/desorption analysis of the powders of DS-1, DS·HCl-1, DS·HCl-2 shows a typical type III isotherm.

1.6. Infrared spectra and UV-vis spectra of PANI

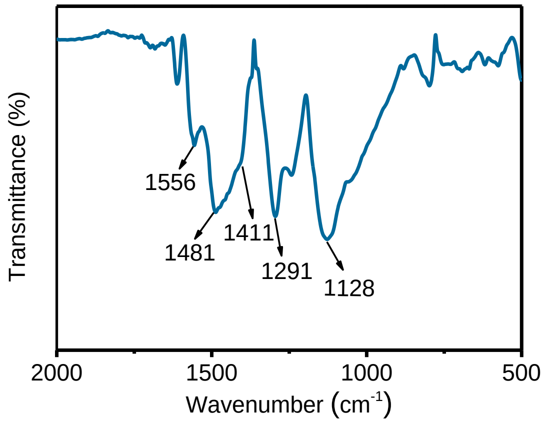

Figure S47. The Infrared spectra of PANI. Related to STAR Methods.

For PANI, the nitrogen quinone group (N=Q=N) stretching vibration at 1556 cm^{-1} and the nitrogen benzene group (N-B-N) stretching vibration at 1481 cm^{-1}. Besides, PANI exhibit two IR bands at ~1411 cm^{-1} and at ~1291 cm^{-1} resulting from stretching vibration of N=N and stretching vibration of C-N in quinoid-benzenoid units (QBQ, BBQ, QBB)[20,21]. Notably, the 1128 cm^{-1} band is a vibrational mode of B-NH^{+}=Q or B-NH^{+}-B[20]. The infrared spectroscopy demonstrates that PANI is in the conducting emeraldine salt (half-oxidized and protonated form; ES) state.

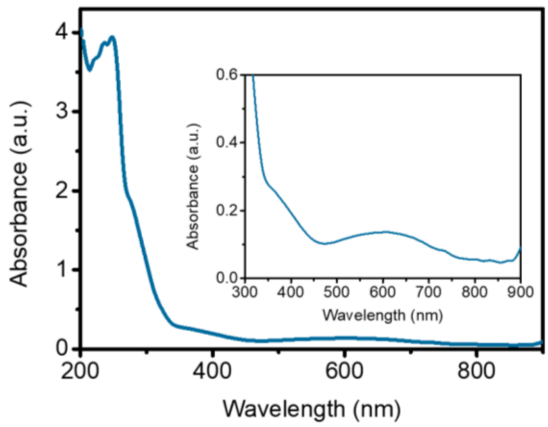

Figure S48. The UV-vis spectra of PANI. Related to STAR Methods.

The shoulder at 278 nm corresponds to the π-π* transition of the benzenoid rings, while the two broad bands around 350-420 nm and 530-710 nm originate from the localized polarons and polaron band-π* band transitions[22,23]. These spectral features are characteristic of the protonated PANI, which represents the conducting ES phase of the polymer[4].

II.Supplemental Tables

Table S1. EIS parameters of different samples obtained by fitting the experimental data to the model shown in Figure. S31A. Related to Figure 5. See text for abbreviations.

Samples	blank	LS-1	LS·HCl-1	LS·HCl-2	PANI	DS·HCl-2	LP+ DP (1:1)
R_{CT} (Ω·cm^2)	24910	5191	1467	571.8	1261	546.4	1248
CPE-C (S·sec^n/ cm^2)	2.525×10^{-5}	5.107×10^{-5}	6.11×10^{-5}	8.802×10^{-5}	6.395×10^{-5}	6.036×10^{-5}	1.651×10^{-5}
CPE-P	0.4969	0.3686	0.5556	0.6382	0.6435	0.3711	0.6124
Warburg (S·sec^5/ cm^2)	2.659×10^{6}	1.27×10^{11}	2.959×10^{8}	0.02165	0.007467	2.882×10^{-4}	1.598×10^{-4}
R_S (Ω·cm^2)	815.7	226.4	448	374.5	175.4	69.94	168.6

Table S2. EIS parameters of LS·HCl-2 and PANI during redox obtained by fitting the experimental data to the model shown in Figure. S31A. Related to Figure 5. See text for abbreviations.

Samples	LS·HCl-2 original	LS·HCl-2 oxidation	LS·HCl-2 reduction	PANI original	PANI oxidation	PANI reduction
R_{CT} (Ω·cm^2)	514.7	379.5	478.7	944.8	3216	1660
CPE-C (S·sec^n/ cm^2)	8.699×10^{-6}	4.575×10^{-6}	2.387×10^{-6}	1.122×10^{-5}	8.766×10^{-6}	4.015×10^{-6}
CPE-P	0.8492	0.774	0.8169	0.8664	0.8202	0.8387
Warburg (S·sec^5/ cm^2)	2.593×10^{-3}	4.581×10^{-4}	2.073×10^{-4}	2.087×10^{-3}	5.719×10^{-4}	7.657×10^{-4}
R_S (Ω·cm^2)	25.1	69.25	104.6	27.13	28.32	30.69

Table S3. Calculation of C content from XPS (Figure S40) C (1s). Related to Figure 5.

Sample	C-C/C-H/C=C	%At Conc	C-N/C=N	%At Conc	C=O/C-O	%At Conc
L-PheAD	284.77 (eV);	68.88	285.96 (eV)	21.89	288.63 (eV);	9.23
PANI	284.78 (eV);	66.83	286.21	9.73	288.64 (eV);	23.44

Table S4. Calculation of N content from XPS (Figure S40) N (1s). Related to Figure 5.

Sample	-N=	%At Conc	-NH-	%At Conc	$-N^+-$	%At Conc
L-PheAD	397.40 (eV);	6.70	400.05 (eV)	76.44	401.09 (eV);	16.86
PANI	397.10 (eV);	8.00	399.99 (eV)	78.48	402.19 (eV);	13.52

Table S5. Calculation of Cl content from XPS (Figure S40) Cl (2p). Related to Figure 5.

Sample	Cl^-	%At Conc	-Cl	%At Conc
L-PheAD	198.66 (eV); 200.26 (eV)	91.12	200.49 (eV); 202.09 (eV)	8.88
PANI	198.66 (eV); 200.26 (eV)	93.58	200.33 (eV); 201.93 (eV)	6.42

Table S6. Comparison of stability of reported electrochemical sensors based on PANI. Related to Figure 6.

Modified electrode	Detection methods	Storage	The retained response current of the sensor after storage	Reference
AuNP–PANI–Gra/GCE	CV	4 °C, >28 days	89.7%	Bai, L. et al., 2013[24]
PANI–Fe_2O_3–rGO/GCE	CV and DPV	In PBS solution at room temperature, >21 days	95.17%	Radhakrishnan, S. et al., 2015[25]
Gra/CuPc/PANI	CV and Amperometric	In 0.1 M PBS solution, >21 days	92.5%	Pakapongpan, S. et al., 2014[26]
PANI/NF	CV and Amperometric	>20 days	> 90%	Liu, K. et al., 2019[27]
Fe_3O_4@PANI/rGO/GCE	CV and Amperometric	In 100 μM H_2O_2/0.5 M KOH, >25 days	78.1%	Gabunada, J. C. et al., 2019[28]
PANI-PMMA/ITO	CV	room temperature, >17 days	-	Simsek, M. et al., 2019[29]
Cu-HNT/PANI/GCE	CV and Amperometric	room temperature, >15 days	80%	Luo, Y. et al., 2021[30]
PANI/AuNP/GCE	CV and DPV	room temperature, >1 days	98%	Huang, S. et al., 2021[31]
AuNP/PANI-MWCNTs	Stripping voltammograms	room temperature, >30 days	-	Shao, Y. et al., 2021[32]
DS·HCl-CHIT/GCE (This work)	DPV	room temperature, >60 days	93%	This work

Abbreviations: Gra, Graphene; AuNP, Gold nanoparticle; rGO, Reduced graphene oxide; CuPc, Copper (II) phthalocyanine-tetrasulfonic; NF, Nickel foam; PMMA, Poly(methyl methacrylate); Cu-HNT, Cu nanoparticles decorated halloysite nanotube; MWCNTs, Multi-walled carbon nanotubes; CHIT, Chitosan.

Table S7. Performance comparisons of the reported glutamate biosensors. Related to Figure 6.

Modified electrode	Immo[a]	Detection methods	Linear range (μM)	Sensitivity(μAmM^{-1}cm^{-2})	Storage	Reference
GLDH/VACNTs/SiW	cross-linking	DPV	0.1-500	182	4 °C, >14 days	Gholizadeh, A. et al., 2012[33]
GluOx/C-dot-AgNP-Lum/GCE	Adsorpti-on	Electrochemiluminescence (ECL)	5-5000	-	4 ℃	Zhu, S. et al., 2017[34]
GluOx/CHIT-CNT-AuNW/GCE	cross-linking	Flow amperometry (FA)	5-20000	1.876×10^{-3}	4 °C, >16 days	Kitikul, J. et al., 2017[35]
ZnO NFs/rGO/μPAD	-	photocurrent intensity	2×10^{-5}-1	-	-	Kong, Q. et al., 2018[36]
GluOx/ppy/Pt-MWCNTs/GCE	Adsorpti-on	Amperometric	10-100	723.08	−20 °C or 4 °C, 30 days	Maity, D. et al., 2019[37]
GluOx/Co_3O_4 nanocubes/SPE	cross-linking	Amperometric	10-600	20.12	4 ℃, 30 days	Hu, F. et al., 2020[38]
GluBP/AuNP/SPCE	-	CV	0.1-0.8	-	-	Zeynaloo, E. et al., 2021[39]
DS·HCl-CHIT/GCE (This work)	-	DPV	25-1200	254.95	room tempera-ture, >60 days	This work

[a] Immobilization methods

Abbreviations: GLDH, Glutamic dehydrogenase; VACNT, Vertically aligned carbon nanotubes; SiW, Silicon wafer; GluOx, Glutamate oxidase; AgNP, Ag nanoparticles; Lum, Luminol; CHIT, Chitosan; CNT, carbon nanotube; AuNW, gold nanowire; ZnO NFs, zinc oxide nanoflowers; μPAD, microfluidic paper-based device; SPE, screen-printed electrode; GluBP, glutamate binding protein; AuNP, gold nanoparticle; SPCE, screen-printed carbon electrode

Table S8. Determination of L/D-Glu in horse serum samples. Related to Figure 6. Recovery experiments were performed by spiking horse serum samples with different amounts of L/D-Glu to explore the biological applications of DS·HCl-2 fabricated sensors. According to the linear regression equations, the average recoveries of L-Glu and D-Glu in serum were 89.8% and 100.8% respectively.

Sample	Added L-Glu (μM)	Found L-Glu (μM)	Recovery (%)	Added D-Glu (μM)	Found D-Glu (μM)	Recovery (%)
1	50	43.8	87.6	50	47.3	94.6
2	50	46.0	92.0	50	53.5	107

REFERENCES

1. Albert, R. (1963). Concepts in photoconductivity and allied problems (Interscience Publishers).
2. Li, D., Lan, C., Manikandan, A., Yip, S., Zhou, Z., Liang, X., Shu, L., Chueh, Y.-L., Han, N., and Ho, J.C. (2019). Ultra-fast photodetectors based on high-mobility indium gallium antimonide nanowires. Nat. Commun. *10*, 1664. https://doi.org/10.1038/s41467-019-09606-y.
3. Fears, K.P., Kolel-Veetil, M.K., Barlow, D.E., Bernstein, N., So, C.R., Wahl, K.J., Li, X., Kulp III, J.L., Latour, R.A., and Clark, T.D. (2018). High-performance nanomaterials formed by rigid yet extensible cyclic β-peptide polymers. Nat. Commun. *9*, 4090. https://doi.org/10.1038/s41467-018-06576-5.

4. Zhang, W.J., Feng, J., Macdiarmid, A.G., and Epstein, A.J. (1997). Synthesis of oligomeric anilines. Synth. met. *84*, 119-120. https://doi.org/10.1016/S0379-6779(97)80674-1.
5. Nalwa, H.S. (1991). Structural determination of a semiconductive tetramer of aniline by IR, UV-visible, ESR, XPS and mass spectroscopy techniques. J. Mater. Sci. *26*, 1683-1690. https://doi.org/10.1007/BF00544683.
6. Wei, Z., Laitinen, T., Smarsly, B., Ikkala, O., and Faul, C.F.J. (2005). Self-Assembly and Electrical Conductivity Transitions in Conjugated Oligoaniline-Surfactant Complexes. Angew. Chem. Int. Ed. *117*, 761-766. https://doi.org/10.1002/anie.200460928.
7. Surwade, S.P., Manohar, N., and Manohar, S.K. (2009). Origin of Bulk Nanoscale Morphology in Conducting Polymers. Macromolecules *42*, 1792-1795. https://doi.org/10.1021/ma900141g.
8. Wang, Y., Tran, H.D., Liao, L., Duan, X., and Kaner, R.B. (2010). Nanoscale morphology, dimensional control, and electrical properties of oligoanilines. J. Am. Chem. Soc. *132*, 10365-10373. https://doi.org/10.1021/ja1014184.
9. Shao, Z., Rannou, P., Sadki, S., Fey, N., Lindsay, D.M., and Faul, C.F.J. (2011). Delineating poly(aniline) redox chemistry by using tailored oligo(aryleneamine)s: towards oligo(aniline)-based organic semiconductors with tunable optoelectronic properties. Chem. Eur. J. *17*, 12512-12521. https://doi.org/10.1002/chem.201101697.
10. Shao, Z., Yu, Z., Hu, J., Chandrasekaran, S., Lindsay, D.M., Wei, Z., and Faul, C.F.J. (2012). Block-like electroactive oligo(aniline)s: anisotropic structures with anisotropic function. J. Mater. Chem. *22*, 16230-16234. https://doi.org/10.1039/C2JM32278A.
11. Lv, W., Feng, J., Yan, W., and Faul, C.F.J. (2014). Self-assembly and pH response of electroactive liquid core-tetra(aniline) shell microcapsules. J. Mater. Chem. B *2*, 4720-4725. https://doi.org/10.1039/C4TB00398E.
12. Bell, O.A., Wu, G., Haataja, J.S., Brommel, F., Fey, N., Seddon, A.M., and Harniman, R.L. (2015). Self-Assembly of a Functional Oligo(Aniline)-Based Amphiphile into Helical Conductive Nanowires. J. Am. Chem. Soc. *137*, 14288-14294. https://doi.org/10.1021/jacs.5b06892.
13. Dong, S.L., Han, L., Du, C.X., Wang, X.Y., Li, L.H., and Wei, Y. (2017). 3D Printing of Aniline Tetramer-Grafted-Polyethylenimine and Pluronic F127 Composites for Electroactive Scaffolds. Macromol. Rapid. Commun. *38*, 1600551. https://doi.org/10.1002/marc.201600551.
14. Arukula, R., Thota, A., Boga, K., Narayan, R., and Rao, C.R.K. (2018). Investigations on anticorrosive, thermal, and mechanical properties of conducting polyurethanes with tetraaniline pendent groups. Polym. Advan. Technol. *29*, 1620-1631. https://doi.org/10.1002/pat.4267.
15. Guo, B., Qu, J., Zhao, X., and Zhang, M. (2019). Degradable conductive self-healing hydrogels based on dextran-graft-tetraaniline and N-carboxyethyl chitosan as injectable carriers for myoblast cell therapy and muscle regeneration. Acta Biomater. *84*, 180-193. https://doi.org/10.1016/j.actbio.2018.12.008.
16. Wu, S., and Han, X. (2005). Study on the electrochemical degradation of poly-N-acetylaniline by electro-hydrolysis and electro-chlorination. Polymer Degradation and Stability *90*, 535-539. https://doi.org/10.1016/j.polymdegradstab.2005.04.002.
17. Kumar, S.N., Gaillard, F., Bouyssoux, G., and Sartre, A. (1990). High-resolution XPS

studies of electrochemically synthesized conducting polyaniline films. Synthetic Metals *36*, 111-127. https://doi.org/10.1016/0379-6779(90)90240-L.

18. Wei, Y., Tarekegne, A.T., and Ou, H. (2018). Influence of negative-U centers related carrier dynamics on donor-acceptor-pair emission in fluorescent SiC. Journal of Applied Physics *124*, 054901. 10.1063/1.5037167. https://doi.org/10.1063/1.5037167.
19. Wang, C., Ma, L., Wang, S., and Zhao, G. (2021). Efficient Photoluminescence of Manganese-Doped Two-Dimensional Chiral Alloyed Perovskites. The Journal of Physical Chemistry Letters *12*, 12129-12134. https://doi.org/10.1021/acs.jpclett.1c03583.
20. Tang, J.S., Jing, X.B., Wang, B.C., and Wang, F.S. (1988). Infrared spectra of soluble polyaniline. Synth. Met. *24*, 231-238. https://doi.org/10.1016/0379-6779(88)90261-5.
21. Abdiryim, T., Zhang, X.G., and Jamal, R. (2005). Comparative studies of solid-state synthesized polyaniline doped with inorganic acids. Mater. Chem. Phys. *90*, 367-372. https://doi.org/10.1016/j.matchemphys.2004.10.036.
22. Huyen, D.N., Tung, N.T., Thien, N.D., and Thanh, L.H. (2011). Effect of TiO2 on the Gas Sensing Features of TiO2/PANi Nanocomposites. Sensors *11*, 1924-1931. https://doi.org/10.3390/s110201924.
23. Yang, D., Lu, W., Goering, R., and Mattes, B.R. (2009). Investigation of polyaniline processibility using GPC/UV–vis analysis. Synth. Met. *159*, 666-674. https://doi.org/10.1016/j.synthmet.2008.12.013.
24. Bai, L., Yan, B., Chai, Y., Yuan, R., Yuan, Y., Xie, S., Jiang, L., and He, Y. (2013). An electrochemical aptasensor for thrombin detection based on direct electrochemistry of glucose oxidase using a functionalized graphene hybrid for amplification. Analyst *138*, 6595-6599. https://doi.org/10.1039/C3AN00983A.
25. Radhakrishnan, S., Krishnamoorthy, K., Sekar, C., Wilson, J., and Kim, S.J. (2015). A promising electrochemical sensing platform based on ternary composite of polyaniline–Fe_2O_3–reduced graphene oxide for sensitive hydroquinone determination. Chem. Eng. J. *259*, 594-602. https://doi.org/10.1016/j.cej.2014.08.047.
26. Pakapongpan, S., Mensing, J.P., phokharatkul, D., Lomas, T., and Tuantranont, A. (2014). Highly selective electrochemical sensor for ascorbic acid based on a novel hybrid graphene-copper phthalocyanine-polyaniline nanocomposites. Electrochimica Acta *133*, 294-301. https://doi.org/10.1016/j.electacta.2014.03.167.
27. Liu, K., Duan, X., Yuan, M., Xu, Y., Gao, T., Li, Q., Zhang, X., Huang, M., and Wang, J. (2019). How to fit a response current-concentration curve? A semi-empirical investigation of non-enzymatic glucose sensor based on PANI-modified nickel foam. Journal of electroanalytical chemistry *840*, 384-390. https://doi.org/10.1016/j.jelechem.2019.04.018.
28. Gabunada, J.C., Vinothkannan, M., Kim, D.H., Kim, A.R., and Yoo, D.J. (2019). Magnetite Nanorods Stabilized by Polyaniline/Reduced Graphene Oxide as a Sensing Platform for Selective and Sensitive Non-enzymatic Hydrogen Peroxide Detection. Electroanalysis *31*, 1507-1516. https://doi.org/10.1002/elan.201900134.
29. Simsek, M., Kruechten, L.V., Buchner, M., Duerkop, A., Baeumner, A.J., and Wongkaew, N. (2019). An efficient post-doping strategy creating electrospun conductive nanofibers with multi-functionalities for biomedical applications. J. of Mater. Chem. C *7*, 9316-9325. https://doi.org/10.1039/C9TC03238J.

30. Luo, Y., Liu, W., Huang, M., Zhang, S., Zhao, Y., Yang, Q., Yan, B., Gu, Y., and Chen, S. (2021). Copper Nanoparticles Decorated Halloysite Nanotube/Polyaniline Composites for High Performance Non-Enzymatic Glucose Sensor. J. Electrochem. Soc. *168*, 086504. https://doi.org/10.1149/1945-7111/ac1b4d.
31. Huang, S., Yang, J., Li, S., Qin, Y., Mo, Q., Chen, L., and Li, X. (2021). Highly sensitive molecular imprinted voltammetric sensor for resveratrol assay in wine via polyaniline/gold nanoparticles signal enhancement and polyacrylamide recognition. J. Electroanal. Chem. *895*, 115455. https://doi.org/10.1016/j.jelechem.2021.115455.
32. Shao, Y., Dong, Y., Bin, L., Fan, L., Wang, L., Yuan, X., Li, D., Liu, X., and Zhao, S. (2021). Application of gold nanoparticles/polyaniline-multi-walled carbon nanotubes modified screen-printed carbon electrode for electrochemical sensing of zinc, lead, and copper. Microchem. J. *170*, 106726. https://doi.org/10.1016/j.microc.2021.106726.
33. Gholizadeh, A., Shahrokhian, S., Zad, A.I., Mohajerzadeh, S., Vosoughi, M., Darbari, S., and Sanaee, Z. (2012). Mediator-less highly sensitive voltammetric detection of glutamate using glutamate dehydrogenase/vertically aligned CNTs grown on silicon substrate. Biosens. Bioelectron. *31*, 110-115. https://doi.org/10.1016/j.bios.2011.10.002.
34. Zhu, S., Lin, X., Ran, P., Mo, F., Xia, Q., and Fu, Y. (2017). A glassy carbon electrode modified with C-dots and silver nanoparticles for enzymatic electrochemiluminescent detection of glutamate enantiomers. Microchim. Acta *184*, 4679-4684. https://doi.org/10.1007/s00604-017-2515-6.
35. Kitikul, J., Satienperakul, S., Preechaworapun, A., Pookmanee, P., and Tangkuaram, T. (2017). A simple flow amperometric electrochemical biosensor based on chitosan scaffolds and gold nanowires modified on a glassy carbon electrode for detection of glutamate in food products. Electroanal. *29*, 264-271. https://doi.org/10.1002/elan.201600263.
36. Kong, Q., Wang, Y., Zhang, L., Xu, C., and Yu, J. (2018). Highly sensitive microfluidic paper-based photoelectrochemical sensing platform based on reversible photo-oxidation products and morphology-preferable multi-plate ZnO nanoflowers. Biosens. Bioelectron. *110*, 58-64. https://doi.org/10.1016/j.bios.2018.03.050.
37. Maity, D., and Kumar, R.T.R. (2019). Highly sensitive amperometric detection of glutamate by glutamic oxidase immobilized Pt nanoparticle decorated multiwalled carbon nanotubes(MWCNTs)/polypyrrole composite. Biosens. Bioelectron. *130*, 307-314. https://doi.org/10.1016/j.bios.2019.02.001.
38. Hu, F., Liu, T., Pang, J., Chu, Z., and Jin, W. Facile preparation of porous Co_3O_4 nanocubes for directly screen-printing an ultrasensitive glutamate biosensor microchip. Sensor. Actuat. B: Chem. *306*, 127587. https://doi.org/10.1016/j.snb.2019.127587.
39. Zeynaloo, E., Yang, Y.-P., Dikici, E., Landgraf, R., Bachas, L.G., and Daunert, S. (2021). Design of a mediator-free, non-enzymatic electrochemical biosensor for glutamate detection. Nanomed: Nanotechnol. Biol. Med. *31*, 102305. https://doi.org/10.1016/j.nano.2020.102305.
